# Downregulation of MLL1 Promotes Intestinal Epithelial Barrier Repair Through Gata4/Bmp4 Activation to Ameliorate Crohn's Disease‐Like Colitis in Mice

**DOI:** 10.1111/cpr.70182

**Published:** 2026-02-24

**Authors:** Xue Song, Jing Li, Yue Chen, Bohan Li, Yi Yang, Xiaofan Guan, Xiaofeng Zhang, Lu Tao, Zhijun Geng, Lugen Zuo, Yueyue Wang, Lian Wang, Jianguo Hu

**Affiliations:** ^1^ Department of Central Laboratory First Affiliated Hospital of Bengbu Medical University Bengbu China; ^2^ Anhui Province Key Laboratory of Basic and Translational Research of Inflammation‐Related Diseases Bengbu China; ^3^ Department of Clinical Laboratory First Affiliated Hospital of Bengbu Medical University Bengbu China; ^4^ Department of Gastrointestinal Surgery First Affiliated Hospital of Bengbu Medical University Bengbu China

**Keywords:** barrier, Crohn's disease, differentiation, Gata4/Bmp4, MLL1

## Abstract

The dysfunctional reconstitution of the intestinal barrier is pivotal in driving the initiation of inflammatory pathogenesis in Crohn's disease (CD), although the exact pathophysiology underlying this phenomenon has yet to be definitively characterised. This study aimed to investigate the role of the histone methyltransferase mixed lineage leukaemia 1 (MLL1) in the development of CD‐like colitis and to elucidate the mechanism by which MLL1 promotes epithelial cell differentiation. Colonic tissue specimens from CD patients and TNBS‐induced murine models were analysed to assess MLL1 expression dynamics. The functional impact of MLL1 on murine colitis modelling CD was systematically investigated through clinical symptom scoring, histopathological profiling and quantitative evaluation of intestinal barrier integrity. The role of MLL1 in promoting epithelial cell differentiation and repairing the intestinal barrier was investigated through immunofluorescence and western blotting. Additionally, potential mechanisms underlying the reparative effects of MLL1 on intestinal barrier function were explored. MLL1 expression was upregulated in colonic tissues from CD patients and TNBS‐induced murine colitis models. In contrast, MLL1 suppression in the TNBS cohort attenuated mucosal inflammation and downregulated pro‐inflammatory cytokine production (IL‐1β, IL‐6, TNF‐α) within the colonic mucosa. Additionally, reduced MLL1 expression increased the differentiation capacity of intestinal epithelial cells, including goblet cells, absorptive cells and tuft cells, and promoted barrier function restoration in injured colons and lipopolysaccharide‐stimulated colonic organoids. MLL1 downregulation activated the Gata4/Bmp4 signalling pathway, which may contribute to the reparative effects of MLL1 on intestinal barrier integrity. Downregulating MLL1 expression promotes intestinal epithelial cell differentiation by activating the Gata4/Bmp4 pathway. These findings elucidate a pathophysiological mechanism wherein MLL1 suppression potentiates intestinal barrier restoration, thereby attenuating colitis severity in murine models. The observed therapeutic efficacy positions MLL1 inhibition presents a novel strategy for CD management.

## Graphical abstract

This study elucidates the pivotal regulatory role and mechanism of MLL1 in inflammatory bowel disease. Experimental data demonstrated elevated MLL1 expression levels in TNBS‐induced murine colitis models, with pharmacological inhibition of MLL1 significantly promoting recovery from CD‐like colitis by accelerating intestinal barrier restoration. Mechanistically, MLL1‐mediated mucosal repair was associated with activation of the Gata4/Bmp4 signalling axis, highlighting its potential as a therapeutic target for inflammatory bowel pathology.

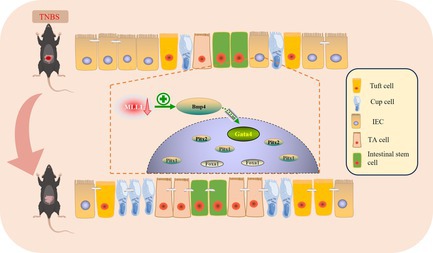

## 1 Introduction

The incidence of Crohn's disease (CD), which is characterised by its chronic, recurrent, debilitating and incurable nature, has increased worldwide [1]. While the exact pathogenesis of CD remains uncertain, the fundamental pathological changes and disease characteristics of CD include damage to the intestinal barrier and dysfunctional repair mechanisms [2]. Recent research has indicated that impaired repair mechanisms of intestinal stem cells (ISCs) are significant factors in the pathogenesis of intestinal barrier dysfunction and colitis in CD [3]. Further investigations in this field are anticipated to offer novel insights into the pathophysiology of the disease and to inform advances in strategies for its clinical management.

The intestinal barrier consists of various components, including mechanical, chemical, immune and biological barriers [4]. Intestinal epithelial cells (IECs) and tight junctions (TJs) form a mechanical barrier that is crucial for preventing harmful substances from entering the body through the intestines [5]. Dysfunction of the intestinal barrier, particularly the mechanical component, can result in the translocation of intestinal bacteria and toxins, potentially leading to the development or worsening of enteritis and impacting postoperative complications and recurrence in patients [6]. Notably, patients with CD may exhibit varying degrees of intestinal barrier dysfunction, including increased intestinal permeability and bacterial translocation, even during periods of remission [7]. Maintaining intestinal barrier integrity represents a critical therapeutic target in CD management. Research indicates that increasing intestinal barrier function via pharmacological methods or gene expression modulation can effectively alleviate CD‐associated enteritis [8, 9, 10]. These findings highlight the importance of not only investigating intestinal barrier dysfunction to better understand the pathogenesis of CD but also addressing intestinal barrier dysfunction as a therapeutic strategy.

IECs play essential roles in forming the intestinal barrier and facilitating nutrient absorption [11]. Physiological damage occurs during the frequent exchange of substances with intestinal contents, leading to the apoptosis and shedding of IECs [12]. ISCs, which are found at the crypt base, typically proliferate and differentiate to replace damaged epithelial cells [13]. This dynamic equilibrium between cell damage and repair is essential for maintaining intestinal homeostasis [14]. However, patients with CD exhibit deficiencies in the repair of intestinal epithelial damage, as indicated by increased numbers of apoptotic cells, defects in the intestinal epithelial structure and decreased intestinal crypt lengths [15]. The compromised structural integrity of the intestinal mucosa allows complex intestinal contents to act as antigens, initiating inflammatory immune responses within the intestines that exacerbate damage to the intestinal barrier [16]. Consequently, impaired intestinal epithelial damage repair has been implicated in the pathogenesis of CD‐related intestinal barrier dysfunction.

Mixed lineage leukaemia 1 (MLL1), which is encoded by the MLL1 gene that is located on chromosome 11 (11q23), is a histone methyltransferase that participates in transcriptional activation and selective gene expression via methylation; MLL1 is crucial for cell proliferation and differentiation [17]. MLL1 is upregulated across diverse inflammatory pathologies and serves as a key regulator of inflammation‐associated tissue injury [18]. Additionally, recent research has demonstrated the significant role of MLL1 in the differentiation of ISCs. Specifically, MLL1 expression is upregulated in actively proliferating ISCs, and its expression decreases and is ultimately lost as ISCs differentiate and mature [19]. MLL1 suppression promotes the differentiation of ISCs into absorptive and goblet cell lineages, implicating its regulatory role in maintaining IEC plasticity [20]. In fact, MLL1 is proposed to inhibit the differentiation of IECs. Furthermore, MLL1 hinders cell differentiation and sustains cell stemness by regulating cell differentiation signals, such as Gata4/Bmp4 [21]. Increased MLL1 expression in response to inflammation suggests its involvement in inhibiting stem cell differentiation; however, its potential role in disordered ISCs differentiation in CD remains uncertain.

This study explored the functional impact of MLL1 on intestinal barrier restoration in CD through integrated in vivo and ex vivo analyses using CD animal models and intestinal organ systems. We investigated the mechanistic role of MLL1 in intestinal epithelial repair, leveraging the differentiation potential of ISCs as a foundational framework. By investigating the MLL1‐Gata4/Bmp4 signalling pathway, the molecular mechanisms underlying the function of MLL1 were analysed in detail. This research aimed to provide insights into the pathophysiological processes of intestinal barrier damage in CD and offer potential guidance for clinical diagnosis and the development of treatment strategies.

## 2 Materials and Methods

### 2.1 Patient Samples

Ethical approval for this study was obtained from the Ethics Committee of Bengbu Medical University (BMU), with informed consent secured from all participants (2024.193). Colonic tissue specimens were acquired from 13 CD patients undergoing initial bowel resection for stenosis and 14 colon cancer patients serving as controls. The CD group included 13 participants (nine male and four female participants) with an average age of 40.8 years (SD = 8.9) and an average BMI of 18.8 kg/m^2^ (SD = 3.5). The control cohort comprised 14 age‐ and sex‐matched colon cancer patients (seven males, seven females; mean age = 48.6 ± 7.0 years; mean BMI = 19.3 ± 2.6 kg/m^2^). Detailed demographic and clinical characteristics are provided in Table S1.

### 2.2 2,4,6‐Trinitrobenzenesulfonic Acid (TNBS)‐Induced Colitis Model

Male C57BL/6J mice (GemPharmatech Co. Ltd., Jiangsu, China) were housed under specific pathogen‐free (SPF) conditions. All experimental protocols received ethical approval from the Institutional Animal Care and Use Committee of BMU (2024.297). Following anaesthesia induction via intraperitoneal administration of 1% pentobarbital (40 mg/kg), animals were positioned supine for intracolonic instillation of 100 μL 2.5% TNBS solution (prepared by 1:1 dilution of 5% TNBS in absolute ethanol). Postadministration, mice remained supine for 5 min to facilitate luminal agent retention [22]. Following TNBS injection, the mice were observed for 1 week and were provided with sufficient water and chow.

### 2.3 Animal Treatments

In total, 50 mice were divided into five groups, namely, the WT, WT (sh‐MLL1), TNBS, TNBS (sh‐MLL1) and (sh‐GATA4) groups, with 10 mice in each group. The mice in the WT group were maintained under standard conditions for 5 weeks. Following a 4‐week acclimatisation period under standard conditions, TNBS‐treated mice received lentiviral delivery of sh‐MLL1 (0.2 mL, 1 × 10^11^ vg) via tail vein injection to achieve MLL1 knockdown, while wild‐type (WT) controls underwent identical procedures. Following a 5‐week acclimatisation under standard conditions, TNBS (sh‐MLL1) group mice were treated with TNBS after a 4‐week postlentiviral administration interval. Similarly, TNBS (sh‐GATA4) group mice received TNBS following sh‐MLL1 or sh‐GATA4 delivery (0.2 mL, 1 × 10^11^ vg, tail vein injection).

### 2.4 Cultivation and Treatment of Colon Organoids

Isolated colonic crypts from WT mice were expanded in IntestiCult medium (STEMCELL, Cat# 06005) for organoid culture [23]. At Day 4 postculture, organoids were randomised into five experimental cohorts: [1] untreated control (NC), [2] NC with MLL1 knockdown (sh‐MLL1), [3] TNF‐α stimulation, [4] TNF‐α + sh‐MLL1 and [5] TNF‐α + sh‐GATA4.

Organoids from lentivirus cultures were dissociated into single‐cell suspensions and seeded at a density of 10^5^ cells per well in 24‐well plates. Following replacement of the original medium with polybrene‐supplemented medium (6 μg/mL), cells were transduced with lentiviral suspension and incubated at 37°C. Postcentrifugation, pelleted cells were resuspended in a Matrigel‐medium mixture and plated into 24‐well culture plates. Puromycin was added to the medium to selectively eliminate nontransfected organoids, allowing for the identification of successfully transfected organoids 3 days posttransplantation [24].

Organoids in the untreated control (NC) group were maintained under standard conditions, while the TNF‐α group was exposed to 20 ng/mL TNF‐α (R&D, Cat #: 410‐MT) for 24 h [25]. Organoids in the sh‐MLL1‐transduced NC group underwent 6‐h lentiviral transduction to achieve MLL1 knockdown, followed by 24‐h culture under standard conditions. The organoids in the TNF‐α (sh‐MLL1) group were subjected to 24 h of stimulation with TNF‐α following incubation with lentivirus. The organoids in the TNF‐α (sh‐GATA4) group were subjected to a 6‐h incubation with lentivirus to selectively downregulate MLL1 and GATA4 gene expression, followed by stimulation with TNF‐α for 24 h. The organoids were collected after the experiment and preserved in either liquid nitrogen or a 10% formalin solution for subsequent analysis.

### 2.5 Body Weight and Disease Activity Index (DAI) Were Monitored Longitudinally to Assess Disease Progression

To evaluate changes in the body weights of the mice, the initial and final weights (after treatment) were measured. Weekly DAI assessments were conducted to quantify the enteritis activity of the mice. The DAI was evaluated using a standardised 6‐point scale (0–5), consistent with established protocols [26].

### 2.6 Collection of Specimens

The mice were euthanised to allow the collection of serum and tissue samples by a researcher who was affiliated with BMU. Colon length was quantified in all mice, followed by systematic harvesting of the colon, mesenteric lymph nodes (MLNs), spleen and liver. Intestinal tissues were longitudinally bisected and fixed in 10% neutral‐buffered formalin or snap‐frozen in liquid nitrogen for downstream analyses.

### 2.7 Fluorescein Isothiocyanate (FITC)

Following a 4‐h fast, mice were orally gavaged with FITC‐dextran (600 mg/kg, 4 kDa; Sigma, Cat #: 46944–100MG‐F) as previously described [27]. Postexperiment, animals were anaesthetised for serum collection, and FITC levels were quantified via fluorometry 4 h postadministration.

### 2.8 Endoscopic Assessment

The mice were anaesthetised with 2%–3% isoflurane before being subjected to endoscopic colitis evaluation. After deep anaesthesia, the endoscope was carefully inserted into the rectum with the activation of the air pump to gradually inflate the colon. Images were captured during the gradual withdrawal of the endoscope. Parameters, such as intestinal wall thickness, vascular morphology, mucosal surface appearance and faecal consistency, were assessed to calculate the endoscopic index of colitis in these mice. Scores ranging from 0 to 15 were used to reflect the severity of colitis, with higher scores indicating increased severity [28].

### 2.9 Transepithelial Electrical Resistance (TEER)

The functional integrity of murine intestinal mucosa was evaluated via Ussing chamber assays (CSYS‐4HA, Warner Instruments) following established protocols [29]. Colonic tissues were flushed with PBS and equilibrated in prewarmed Krebs buffer (35°C–38°C, pH 7.34). Longitudinal strips (3 × 10.5 mm) were mounted on specialised inserts within the precalibrated chamber. The serosal compartment was perfused with Krebs buffer supplemented with 10 mM glucose as an energy substrate. Transepithelial voltage and current were regulated in real time using a computer‐controlled interface (Physiological Systems ACQUIRE/ANALYSE software).

### 2.10 Bacterial Translocation

Paired tissue samples from MLNs, spleens and livers were homogenised after weighing and cultured on MacConkey's agar (Sigma, Cat #: F‐7250) at 37°C for 24 h. Intestinal permeability was evaluated by quantifying bacterial translocation rates across experimental groups [30].

### 2.11 Haematoxylin and Eosin (HE) Staining and Inflammation Assessment

Colonic histopathological alterations were analysed via HE staining, with inflammatory severity graded on a 0–4 scale by two blinded histopathologists [31].

### 2.12 Enzyme‐Linked Immunosorbent Assays (ELISAs)

Mucosal inflammatory cytokine levels (IL‐1β, TNF‐α, IL‐6) were quantified via ELISA (Boster, Cat# EK0394, EK0527, EK0411) following tissue homogenisation in accordance with standardised protocols [32].

### 2.13 Permeability of Organoids to FITC

FITC‐dextran permeability was assessed by confocal microscopy (Zeiss LSM 700, 10× magnification). Organoids were solubilised in 500 μL Cultrex Organoid Harvesting Solution (R&D Systems 3700‐100‐01), incubated at 4°C for 20 min, and centrifuged (500*g*, 5 min). Pelleted cells were resuspended in 30 μL FITC‐dextran (Sigma–Aldrich 46,944‐100 MG‐F), mounted on slides with double‐sided tape to prevent mechanical compression, and imaged [33].

### 2.14 TEER of Organoids

To assess the permeability of the organoid intestinal barrier, TEER was measured. The TEER of the organoids was assessed using the Millicel ERS2 resistance system after the organoids were equilibrated at room temperature (RT) for 20 min. Only measurements that were maintained for 10 s were recorded, and blank measurements were assessed using an empty insert. The TEER value was calculated as the measured value minus the empty insert value multiplied by the area (cm^2^) [34].

### 2.15 Periodic Acid‐Schiff (PAS) Staining

PAS staining was performed on paraffin‐embedded sections, and the tissues were fixed with 75% alcohol. The samples were treated with periodate concentrate and then reacted with Schiff's reagent. The magenta in Schiff's reagent reacts with aldehyde groups to form a reddish precipitate that is localised to the cytoplasm. Differentiation was carried out with 95% ethanol to remove unbound dye, and haematoxylin was used to restain the nuclei [35].

### 2.16 Immunohistochemistry (IHC) Analyses

Deparaffinised and hydrated sections of mouse and human colons were processed for antigen retrieval and the blocking of nonspecific binding sites with goat serum [36]. IHC was performed on colonic tissue samples from both species with antibodies against MLL1 (1:400; CST, Cat#: 14197).

### 2.17 Immunofluorescence Analysis

An immunofluorescence study was performed following a comprehensive review of the literature. Mouse colon sections were deparaffinised, hydrated and subjected to antigen retrieval, and then nonspecific binding was blocked with goat serum [37]. Subsequently, the sections and organoids were incubated with anti‐ZO1, anti‐Claudin1, anti‐MUC2, anti‐Villin, anti‐Dclk1, anti‐Ki67, anti‐Olfm4, anti‐Gata4 and anti‐Bmp4 antibodies at dilutions of 1:300 and 1:500 (Abcam, Cat #: ab307799, ab307692, ab272706, ab97512, ab31704, ab16667 and ab307823; CST, Cat #: 39141S; Proteintech, Cat #: 12492–1‐AP). Fluorescence intensity was measured using laser confocal microscopy (Olympus, FV3000). In each slice, randomly select 10 structurally intact crypts. Ensure that the selected areas are representative and avoid choosing edge or damaged areas [38]. Use image analysis software (ImageJ) to measure the length of each crypt [39], count the number of cells within each crypt [40] and calculate the cell count per millimetre of crypt = (total number of goblet cells in the crypt/total length of the crypt) × 1000 [41].

Fluorescently labelled Ki67 and CgA antibodies were used to identify and quantify TA cells and enteroendocrine cells in colon organoids. By statistically analysing the number of Ki67 or CgA positive cells in 100 organoids, differences between groups were assessed.

### 2.18 Western Blotting Analysis

As previously described, cellular and tissue homogenates were prepared to extract both total protein and membrane proteins; these proteins were subsequently analysed with western blotting techniques [42]. Primary rabbit polyclonal antibodies targeting ZO1, Claudin1, MLL1, Pitx1, Pitx2, Foxa1, Gata4, Bmp4 and β‐actin (at dilutions of 1:600; Abcam, Cat #: ab307799, ab307692, ab221142, ab170933, ab307823 and ab241153; CST, Cat #: 14197; Proteintech, Cat #: 10873‐1‐AP and 12492‐1‐AP) were used. Detection was performed using HRP‐conjugated secondary antibodies (1:3000 dilution; Cell Signalling Technology, Cat #: 7074P2).

### 2.19 qRT–PCR

The transcript levels of IL‐1β, IL‐6 and TNF‐α were determined by RT–qPCR in accordance with the standard protocol [43]. Transcript levels of IL‐1β, IL‐6 and TNF‐α were quantified via RT‐qPCR (Table 1). Total RNA was isolated using TRIzol reagent (Thermo Fisher Scientific, Cat #: 15596026), reverse‐transcribed with a Takara kit (Cat #: RR047), and amplified via PCR (Takara, Cat #: RR820) on a QuantStudio DX system. Relative mRNA expression was normalised to GAPDH and calculated using the 2^−ΔΔC*T*^ method.

**TABLE 1:** Primer sequences (5′3′).

Gene name	Forward primer	Reverse primer
Il‐1β	GCAACTGTTCCTGAACTCAACT	ATCTTTTGGGGTCCGTCAACT
Il‐6	CTGCAAGAGACTTCCATCCAG	AGTGGTATAGACAGGTCTGTTGG
TNF‐α	CAGGCGGTGCCTATGTCTC	CGATCACCCCGAAGTTCAGTAG
GAPDH	AGGTCGGTGTGAACGGATTTG	GGGGTCGTTGATGGCAACA

### 2.20 Statistical Analysis

All statistical analyses were executed by leveraging the capabilities of SPSS v23.0 software, which is developed by SPSS Inc. located in the United States. For datasets that adhered to a normal distribution pattern, the descriptive statistical values were presented in the format of arithmetic means ± standard deviations (SDs). To assess differences across multiple groups, the Analysis of Variance (ANOVA) technique was implemented. For more detailed pairwise comparisons between individual groups, Tukey's post hoc test was specifically chosen. Statistical significance was determined based on a two–tailed *p* value criterion, where a *p* value less than 0.05 was considered to indicate a significant result.

## 3 Results

### 3.1 Elevated MLL1 Levels Were Detected in the Intestinal Mucosa of Not Only Patients With CD But Also TNBS‐Induced Model Mice

**FIGURE 1:**
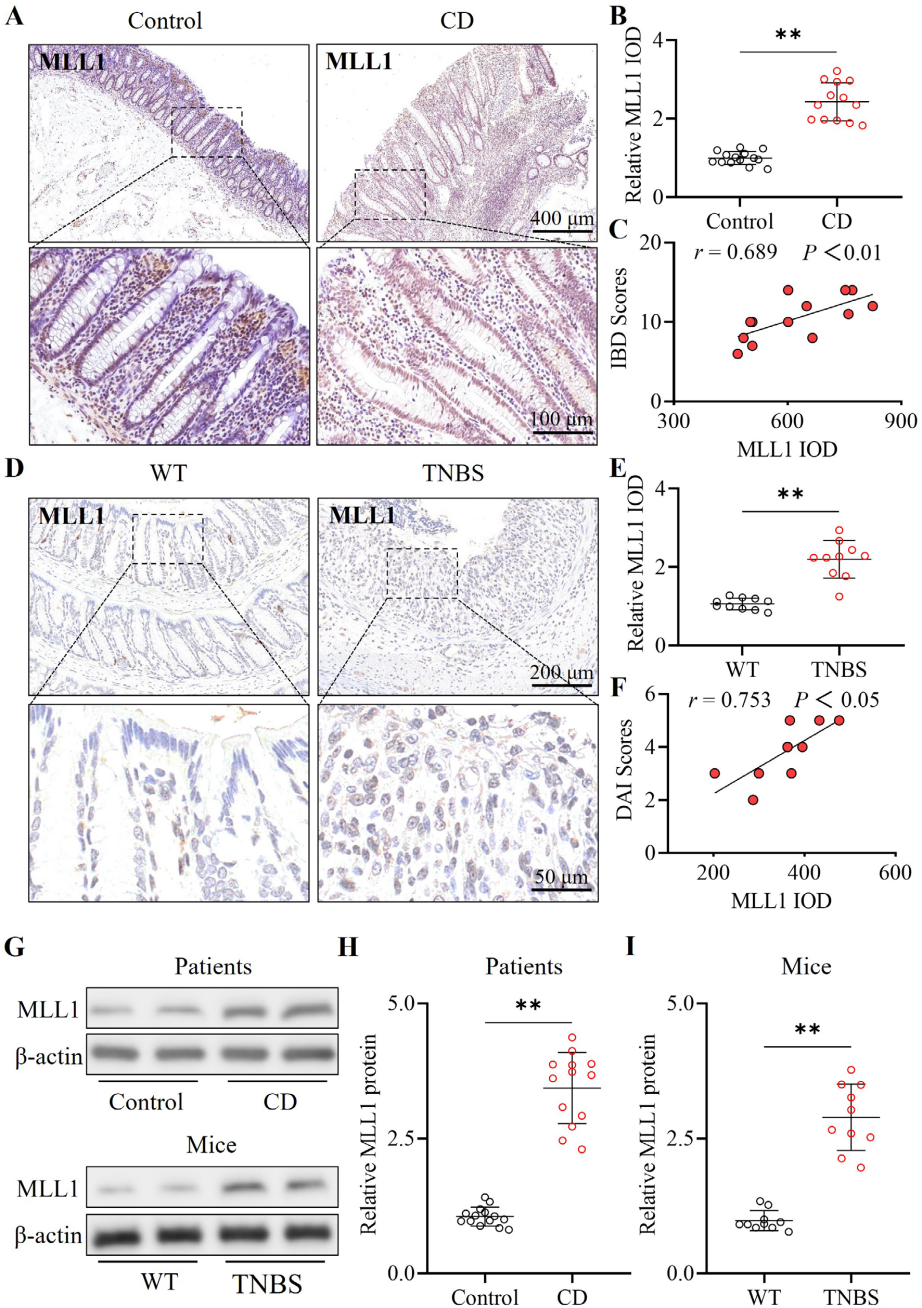
MLL1 upregulation in Crohn's disease (CD) and experimental volitis. (A and B) Immunohistochemical quantification of MLL1 in colonic tissues from CD patients (*n* = 13) versus noninflammatory controls from colon cancer (CRC) patients (*n* = 14). Data represent mean integrated optical density (IOD) ± standard deviation (SD). (C) Correlation analysis of the protein expression of MLL1 and disease activity index of CD patients. (D and E) Comparative IHC analysis of MLL1 expression in wild‐type (WT) mice (*n* = 10) versus TNBS‐induced colitis models (*n* = 10). Results shown as mean IOD ± SD. (F) Correlation analysis of the protein expression of MLL1 and disease activity index of TNBS‐induced. (G–I) Immunoblot analysis of MLL1 protein levels in human and murine colonic mucosa. Relative expression normalised to controls (WT). Statistical significance: *p < 0.05 versus control/WT groups; **p < 0.0001 versus control/WT groups.

Analysis of single‐cell sequencing results from the GEO database (GSE164985) revealed that the expression of MLL1 in colon epithelial cells of CD patients is abnormal compared to the control group (no IBD patients) [44]. Among them, ISCs (ADH1C^+^ Stem cells and STMN1^+^MKI67^+^ Stem cells) and colon absorptive epithelial cells (EAC) showed significant statistical differences between the two groups (*p* < 0.01, Figure S1A–C). In addition, MLL1 is correlated with the gene set of inflammatory response signalling pathways (Figure S1D). Immunohistochemical (IHC) analysis demonstrated significant upregulation and altered spatial distribution of MLL1 in colonic mucosal tissues of CD patients compared to controls (Figure 1A,B). This trend was recapitulated in TNBS‐induced murine colitis models, where MLL1 expression surpassed levels observed in WT mice (Figure 1D). Quantitative IOD measurements confirmed elevated MLL1 expression in TNBS‐treated mice relative to WT counterparts (Figure 1E). Importantly, correlation analysis indicated that MLL1 expression was significantly positively correlated with DAI (Figure 1C,F). Western blotting further corroborated these findings, revealing higher MLL1 protein levels in both CD patient lesions and TNBS‐challenged murine colonic tissues versus healthy controls (Figure 1G–I). Collectively, these data implicate aberrant MLL1 expression in the pathogenesis and progression of CD.

### 3.2 Downregulation of MLL1 Expression Ameliorated CD‐Like Colitis in TNBS‐Treated Mice

**FIGURE 2:**
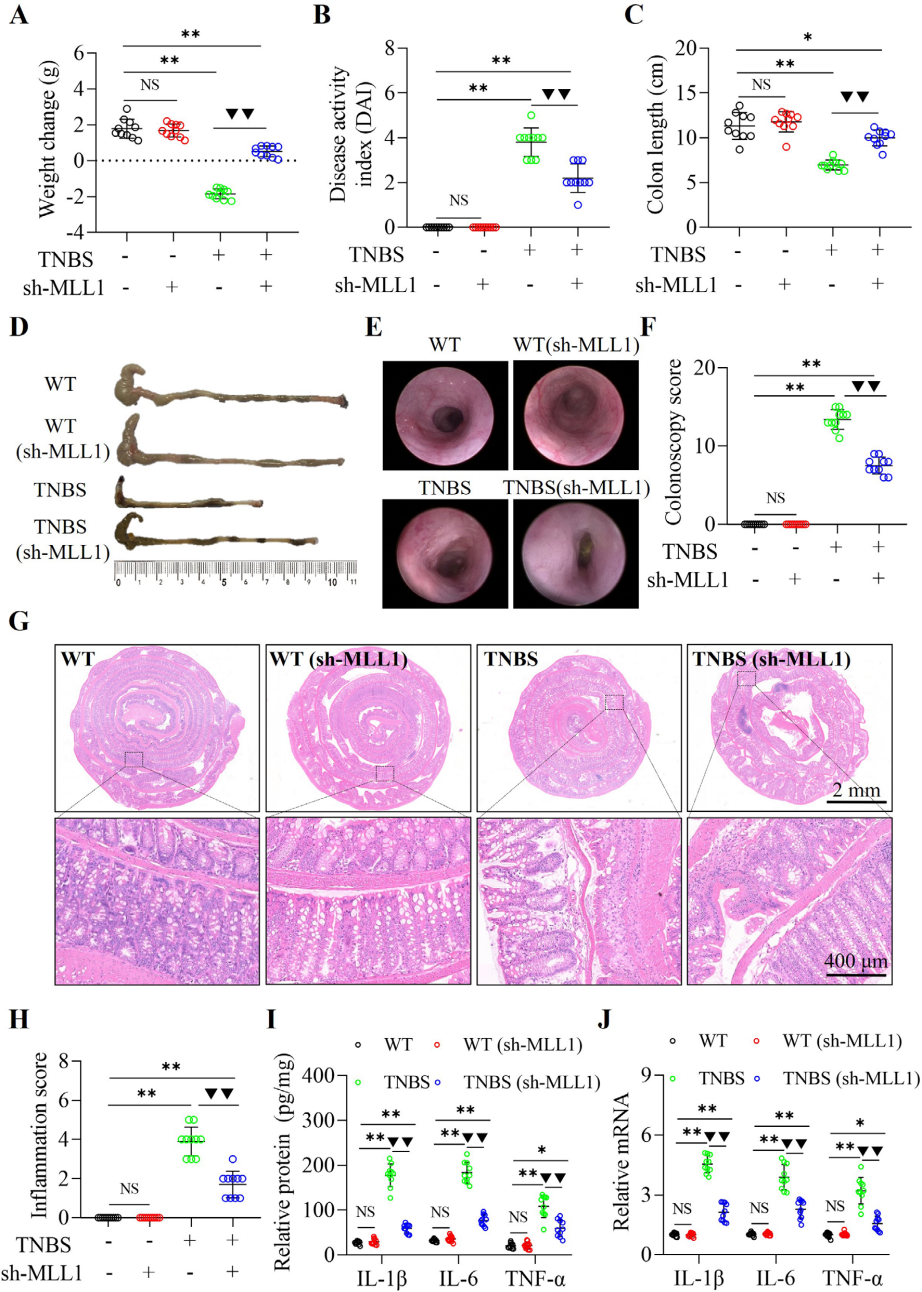
Therapeutic efficacy of MLL1 suppression in TNBS‐driven colitis. (A) Body weight trajectories across experimental groups (*n* = 10/group). (B) Disease activity index (DAI) scoring (*n* = 10/group). (C and D) Colon length measurements (*n* = 10/group). (E and F) Endoscopic severity grading of colonic inflammation (*n* = 10/group). (G) Representative HE‐stained colonic sections. (H) Histopathological inflammation scores (*n* = 10/group). (I) Mucosal IL‐1β/IL‐6/TNF‐α protein levels determined by ELISA (*n* = 10/group). (J) qRT‐PCR quantitation of IL‐1β/IL‐6/TNF‐α mRNA in colonic mucosa (*n* = 10/group). WT (Untreated WT), WT‐shMLL1 (AAV‐mediated MLL1 knockdown), TNBS colitis, TNBS‐shMLL1. Data: Mean ± SD; *p < 0.05 versus WT; ^▼^ *p* < 0.05 versus TNBS; **p < 0.0001 versus WT groups; ^▼▼^ *p* < 0.0001 versus TNBS; NS: No significance, WT (sh‐MLL1) versus WT.

To investigate the role of MLL1 in the pathogenesis of CD, we established a TNBS‐induced colitis model in mice and knocked down MLL1 using AAV‐mediated shRNA delivery. First, the efficiency of MLL1 suppression was validated by qRT‐PCR and Western blotting after tail vein injection of AAV‐shRNA‐MLL1 (Figure S2A–C), while control mice receiving empty AAV vectors showed no significant changes in DAI scores (Figure S2D). Immunofluorescence detection showed that MLL1 was only expressed in a small number of intestinal goblet cells (MUC2^+^), enteroendocrine cells (CgA^+^) and immune cells (CD45^+^), while it was more widely distributed in transit‐amplifying (TA) cells (Ki67^+^) and stem cells (Olfm4^+^). However, in the colon tissue of patients with CD, MLL1 was widely expressed, mainly in ISCs, but was hardly expressed in goblet cells, TA cells, enteroendocrine cells and immune cells, as shown in Figure S1E. Subsequently, phenotypic analyses revealed that MLL1 knockdown alleviated TNBS‐induced weight loss and reduced DAI scores compared to TNBS‐treated controls (Figure 2A,B). Further assessments demonstrated that MLL1 suppression mitigated intestinal inflammation, as evidenced by improvements in colon length, colonoscopic findings and histopathological scores (Figure 2C–H). Consistently, molecular analyses via ELISA and qRT‐PCR confirmed concurrent reductions in mucosal levels of proinflammatory cytokines, including IL‐1β, IL‐6 and TNF‐α (Figure 2I,J). Interestingly, we found that downregulating MLL1 expression in *IL‐10* ^*−/−*^ mice can also effectively improve their symptoms of colitis (Figure S3A–I). These findings establish MLL1 inhibition as a therapeutic strategy for ameliorating colitis phenotypes recapitulating CD pathology.

### 3.3 MLL1 Downregulation Improved Intestinal Barrier Function in TNBS‐Treated Mice

**FIGURE 3:**
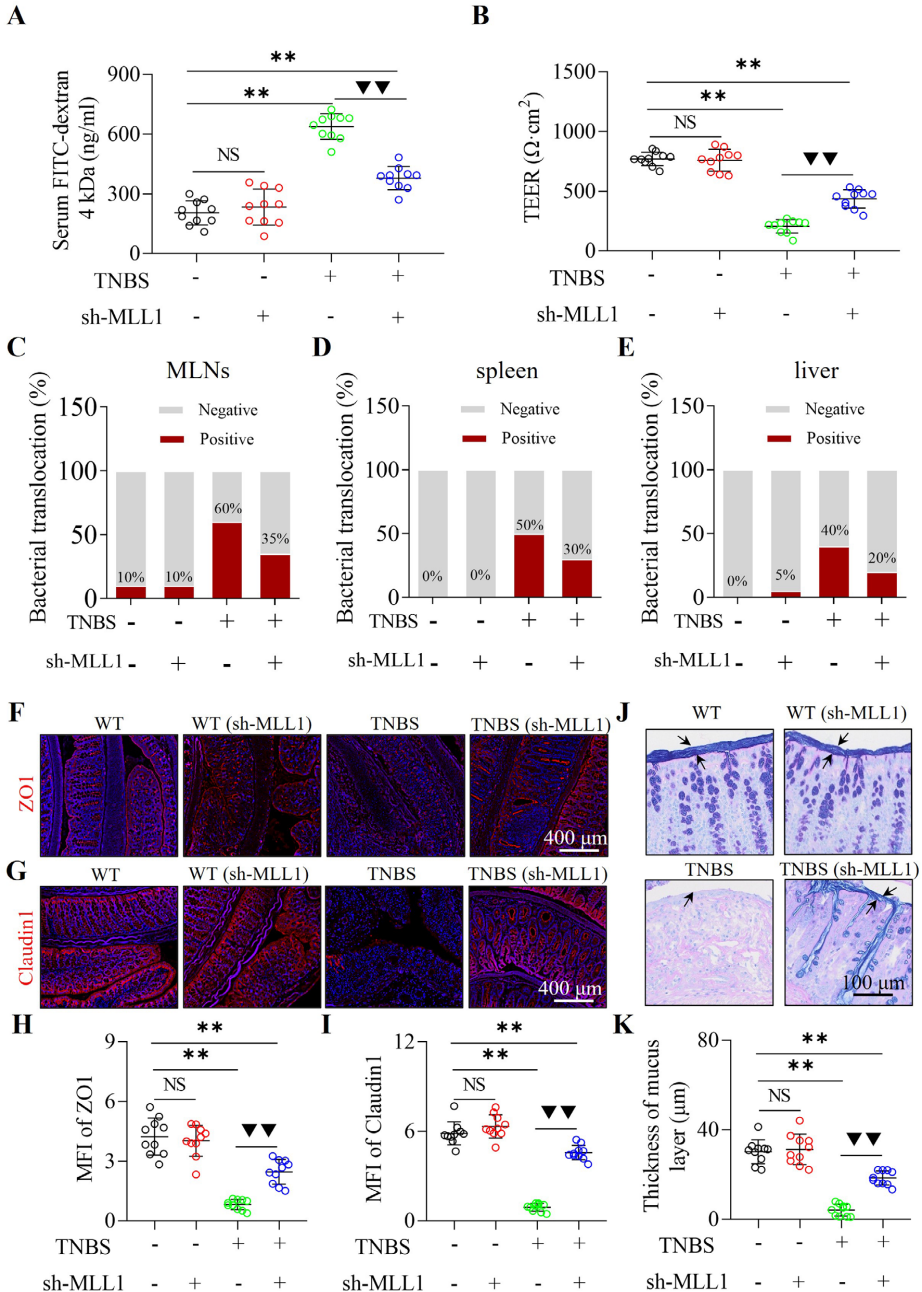
MLL1 knockdown restores intestinal barrier homeostasis in colitis. (A) Serum FITC‐dextran permeability (*n* = 10/group). (B) Transepithelial electrical resistance (TEER) in colonic tissues (*n* = 10/group). (C–E) Bacterial translocation rates to mesenteric lymph nodes (MLNs), spleen, and liver (*n* = 20/group). (F and G) ZO1/Claudin1 localisation via fluorescence microscopy. (H and I) Standardised quantification of mean fluorescence intensity based on immunofluorescence (*n* = 10/group). (J and K) Mucus layer thickness assessed by periodic acid‐Schiff (PAS) staining (*n* = 10/group). Data: Mean ± SD; *p < 0.05 versus WT; ^▼^ *p* < 0.05 versus TNBS; **p < 0.0001 versus WT groups; ^▼▼^ *p* < 0.0001 versus TNBS; NS: No significance, WT (sh‐MLL1) versus WT.

The maintenance of intestinal epithelial integrity has been established as a key determinant in alleviating colitis progression [45]. To clarify the mechanistic role of MLL1 in regulating the intestinal barrier, we utilised TNBS‐induced colitis models with genetically modulated MLL1 expression. Notably, under TNBS challenge, MLL1‐knockdown mice displayed significant reinforcement of the intestinal barrier—this was evidenced by enhanced paracellular sealing capacity (Figure 3A) and improved bioelectric resistance parameters (Figure 3B). Furthermore, this barrier protection effectively inhibited systemic bacterial dissemination, as indicated by colony count analyses showing a marked reduction in bacterial load in the MLNs, spleens and livers compared to the WT TNBS model (Figure 3C–E). Molecular feature analysis reveals that MLL1 regulation generates barrier effects at multiple levels: immunofluorescence results show a significant increase in the continuity of ZO1 and Claudin1 at TJs (Figure 3F–I), this phenomenon was also observed in the colon mucosa of *IL‐10* ^*−/−*^ mice (Figure S3I,J), while histological analysis revealed a significant increase in mucosal mucin content through PAS staining (Figure 3J,K). These multiparametric findings establish that MLL1 suppression coordinates a strategic barrier restoration in experimental CD pathogenesis. This investigation reveals a previously underappreciated regulatory role of MLL1 in epithelial barrier homeostasis during chronic intestinal inflammation.

### 3.4 MLL1 Downregulation Promoted the Differentiation of ISCs in Mouse Colons

**FIGURE 4:**
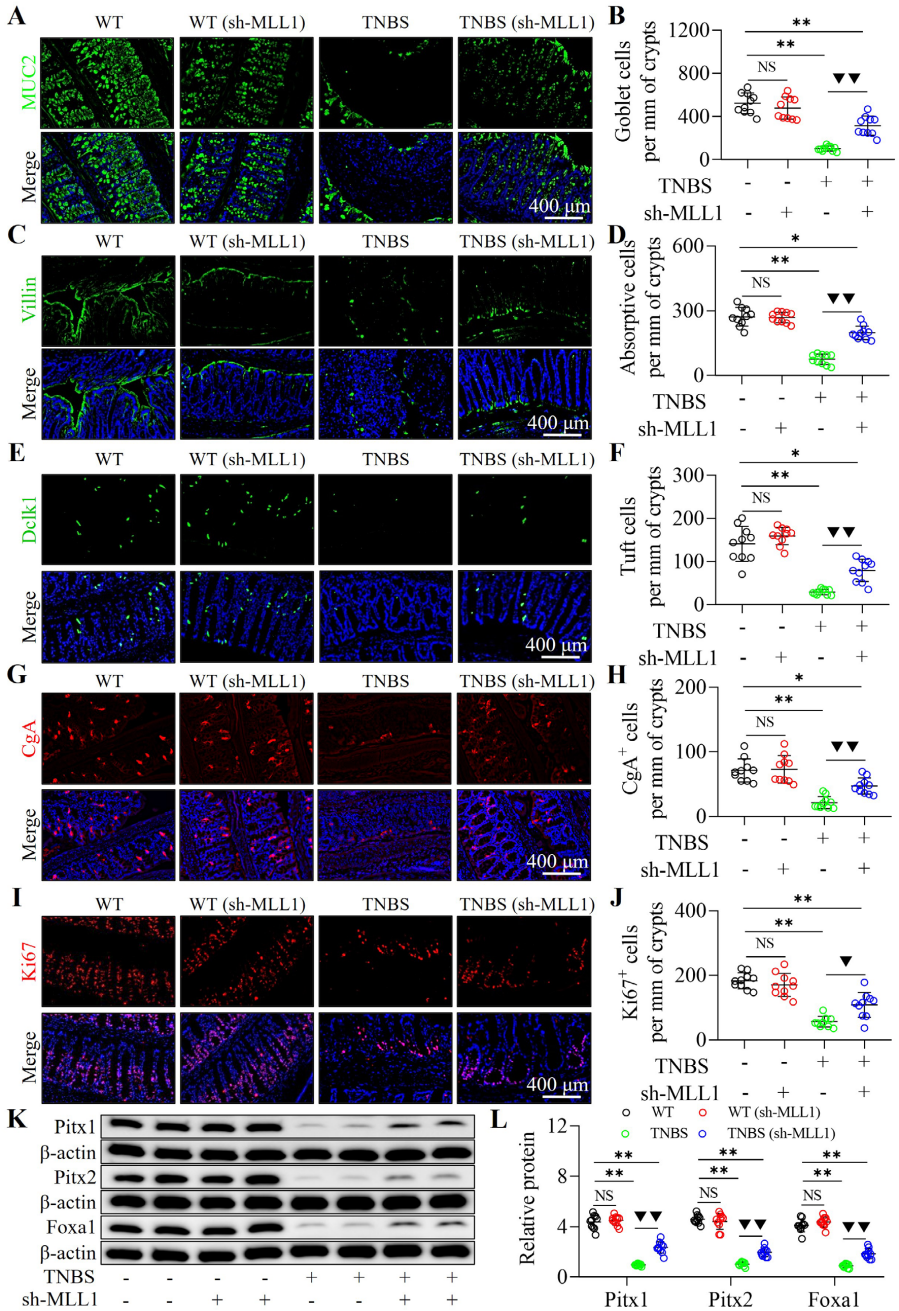
Enhanced epithelial differentiation following MLL1 inhibitions. (A and B) Goblet cell enumeration via MUC2 immunofluorescence (*n* = 10/group). (C and D) Absorptive cell quantification using Villin staining (*n* = 10/group). (E and F) Tuft cell populations identified by Dclk1 fluorescence microscopy (*n* = 10/group). (G and H) Enteroendocrine cells counts via Chromogranin A (CgA) immunostaining (*n* = 10/group). (I and J) Transit‐amplifying (TA) cell counts via Ki67 immunostaining (*n* = 10/group). (K and L) Pitx1/Pitx2/Foxa1 protein levels analysed by immunoblotting (*n* = 10/group). Data: Mean ± SD; *p < 0.05 versus WT; ^▼^ *p* < 0.05 versus TNBS; **p < 0.0001 versus WT groups; ^▼▼^ *p* < 0.0001 versus TNBS; NS: No significance, WT (sh‐MLL1) versus WT.

Emerging evidence highlights enhanced ISCs differentiation potential as a critical mediator of epithelial barrier regeneration [46]; building on these mechanistic insights, we systematically investigated MLL1's regulatory influence on ISCs lineage commitment dynamics. MLL1 downregulation in TNBS‐colitis mice triggered goblet cells hyperplasia within the intestinal mucosa, promoting mucosal barrier reconstitution through amplified secretory activity (Figure 4A,B). Similarly, decreased MLL1 levels also facilitated the capacity of ISCs to differentiate into various forms of IECs [47]. Histomorphometric quantification revealed coordinated epithelial lineage specification in MLL1‐deficient intestinal mucosae, with TNBS‐challenged mice exhibiting expanded absorptive cell (Figure 4C,D), tuft cell compartments (Figure 4E,F) and enteroendocrine cells (Figure 4G,H). Crypt hyperproliferation was evidenced by heightened Ki67^+^ cell densities (Figure 4I,J), suggesting amplified TA progenitor output. Despite equivalent ISCs reservoir sizes between cohorts (Figure S4A,B), MLL1 suppression activated a differentiation‐promoting transcriptional network evidenced by Pitx1/Pitx2/Foxa1 upregulation (Figure 4K,L), which collectively drove barrier restitution through enhanced ISCs‐derived epithelial maturation.

### 3.5 Inhibition of MLL1 Promoted Intestinal Barrier Function in TNF‐α‐Treated Colonic Organoids From Mice

**FIGURE 5:**
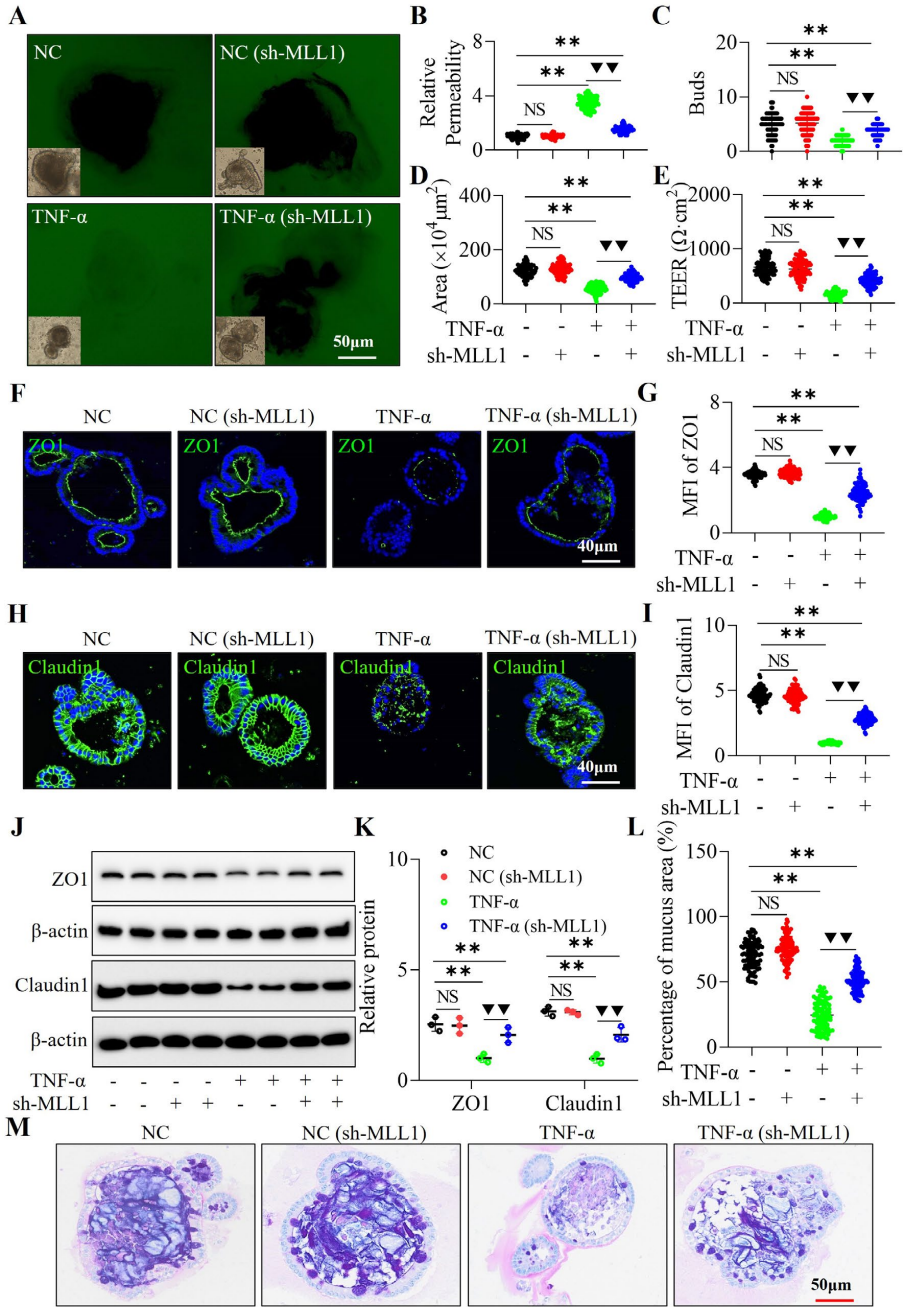
MLL1 suppression mitigates barrier dysfunction in TNF‐α‐stimulated organoids. (A and B) Intraluminal FITC‐dextran accumulation, the image in the bottom right corner is a representative white light microscopy image of organoids (*n* = 100/group). (C and D) The size and buds of the organoids in each group (*n* = 100/group). (E) TEER measurements in colonoid cultures. (F and G) ZO1/Claudin1 distribution via fluorescence microscopy (*n* = 100/group). (H and I) Standardised quantification of mean fluorescence intensity (MFI) based on immunofluorescence (*n* = 100/group). (J and K) Immunoblot analysis of ZO1/Claudin1 protein levels (*n* = 3/group). (L and M) PAS‐based mucus thickness evaluation (*n* = 100/group). Untreated controls (NC), NC‐shMLL1 (AAV‐transduced), TNF‐α‐stimulated, TNF‐α‐shMLL1. Data: Mean ± SD; *p < 0.05 versus NC; ^▼^ *p* < 0.05 versus TNF‐α; **p < 0.0001 versus NC groups; ^▼▼^ *p* < 0.0001 versus TNF‐α; NS: No significance, NC (sh‐MLL1) versus NC.

Several studies have demonstrated that intestinal organoids are reliable in vitro models for investigating intestinal barrier functionality [48, 49]. Lentiviral‐delivered MLL1 knockdown in intestinal organoids induced transcriptomic (qRT‐PCR) and proteomic (western blot) silencing of the target gene (Figure S5A–C). The incubation of TNF‐α‐treated organoids with an empty lentiviral vector did not alter the distribution of FITC‐dextran within the organoid lumen or the TEER value (Figure S5D,E). However, in TNF‐α‐treated organoids, MLL1 expression was reduced, resulting in decreased intestinal barrier permeability. This was shown by the decreased FITC‐dextran distribution in the organoid lumens (Figure 5A,B), increased area and buds of the organoid (Figure 5C,D). Furthermore, monitor TEER values daily to determine the establishment of the colon organoid intestinal barrier model (Figure S5F), and discovering that downregulating MLL1 expression can increase the TEER values of TNF‐α‐treated organoids. (Figure 5E). Moreover, immunofluorescence analysis revealed that MLL1 downregulation effectively reversed the reduction in ZO1 (Figure 5F,G) and Claudin1 (Figure 5H,I) expression in TNF‐α‐treated colonic organoids, and western blotting confirmed these findings (Figure 5J,K). Furthermore, PAS staining of colonic organoids provided further support for the observation that decreased MLL1 expression promoted mucous layer recovery, as shown in Figure 5L,M. These findings further confirm the protective role of MLL1 in restoring intestinal barrier function.

### 3.6 Downregulation of MLL1 Expression Promoted the Differentiation of ISCs in Colonic Organoids

**FIGURE 6:**
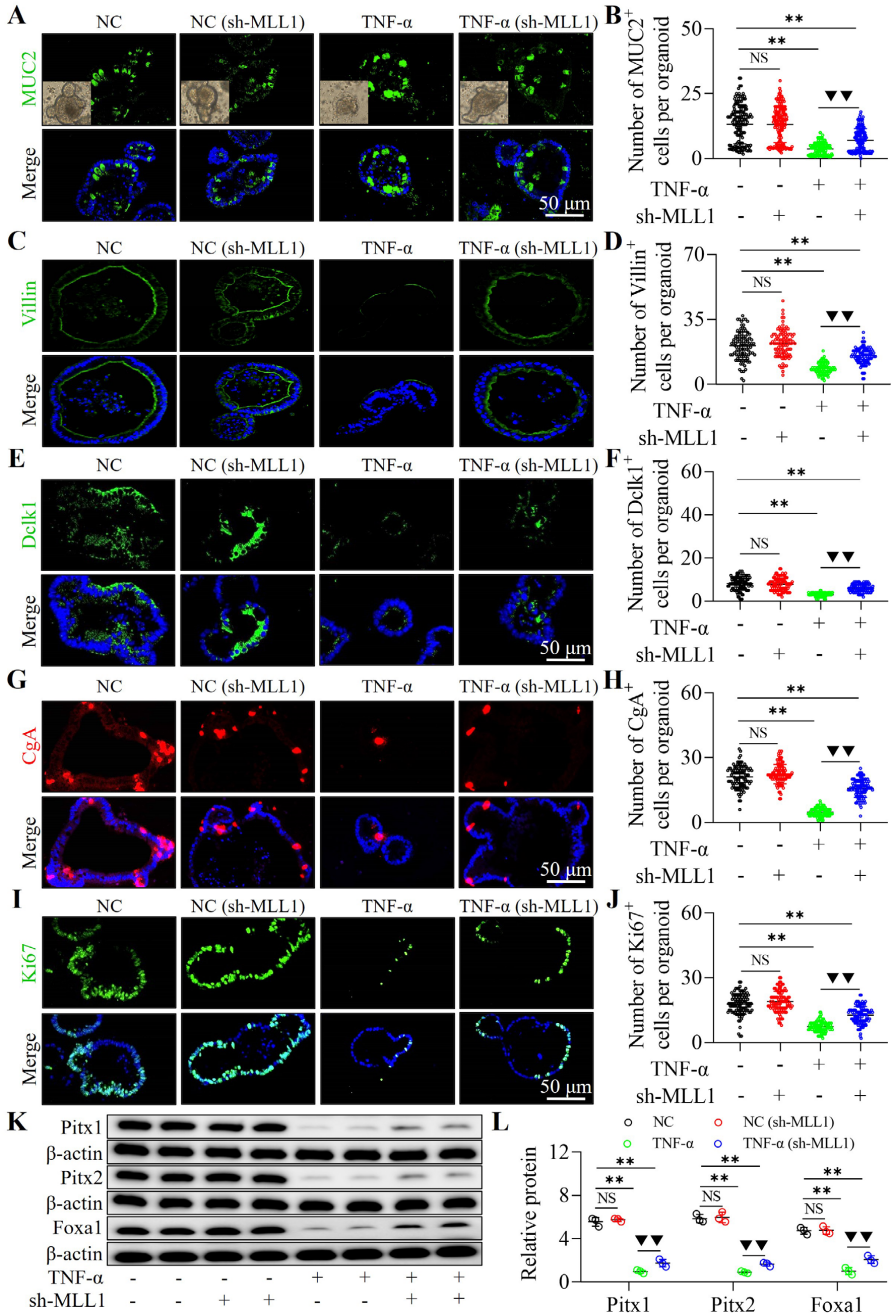
MLL1 knockdown potentiates epithelial maturation in inflamed colonoids. (A and B) The mean fluorescence intensity of MUC2^+^ goblet cell, the image in the bottom right corner is a representative white light microscopy image of organoids (*n* = 100/group). (C and D) The mean fluorescence intensity of Villin^+^ absorptive cell densities (*n* = 100/group). (E and F) The mean fluorescence intensity of Dclk1^+^ tuft cell enumeration (*n* = 100/group). (G and H) CgA^+^ enteroendocrine cell frequencies (*n* = 100/group). (I and J) Ki67^+^ TA cell populations (*n* = 100/group). (K and L) Pitx1/Pitx2/Foxa1 protein profiling by immunoblotting (*n* = 3/group). Data: Mean ± SD; *p < 0.05 versus NC; ^▼^ *p* < 0.05 versus TNF‐α; **p < 0.0001 versus NC groups; ^▼▼^ *p* < 0.0001 versus TNF‐α; NS: No significance, NC (sh‐MLL1) versus NC.

To delineate MLL1's mechanistic involvement in intestinal barrier restoration through ISCs lineage commitment, we conducted systematic differentiation assays using colonic organoid models. Figure 6A,B shows that reducing MLL1 expression in TNF‐α‐treated organoids led to increased goblet cell numbers. MLL1 suppression in TNF‐α (sh‐MLL1)‐treated organoids significantly enhanced the expansion of absorptive cells (Figure 6C,D), tuft cell populations (Figure 6E,F) and enteroendocrine cells (Figure 6G,H) relative to TNF‐α‐stimulated controls. Additionally, our research revealed that decreased MLL1 expression led to an increased abundance of Ki67‐positive cells in TNF‐α‐treated organoids (Figure 6I,J). Notably, MLL1 knockdown failed to alter ISCs quantities in TNFα‐stimulated organoids (TNFα‐shMLL1 vs. TNFα‐CTRL, Figure S6A,B), suggesting lineage commitment is decoupled from stem cell pool maintenance under inflammatory challenge. Correspondingly, the western blotting results revealed that decreased MLL1 expression in TNF‐α‐treated organoids led to increased expression levels of Pitx1, Pitx2 and Foxa1, as shown in Figure 6K,L. These findings indicated a strong correlation between the pro‐differentiation effect of MLL1 and the restoration of intestinal barrier function.

### 3.7 MLL1‐Dependent Suppression of IEC Differentiation Coincided With Epigenetic Silencing of Gata4/Bmp4

**FIGURE 7:**
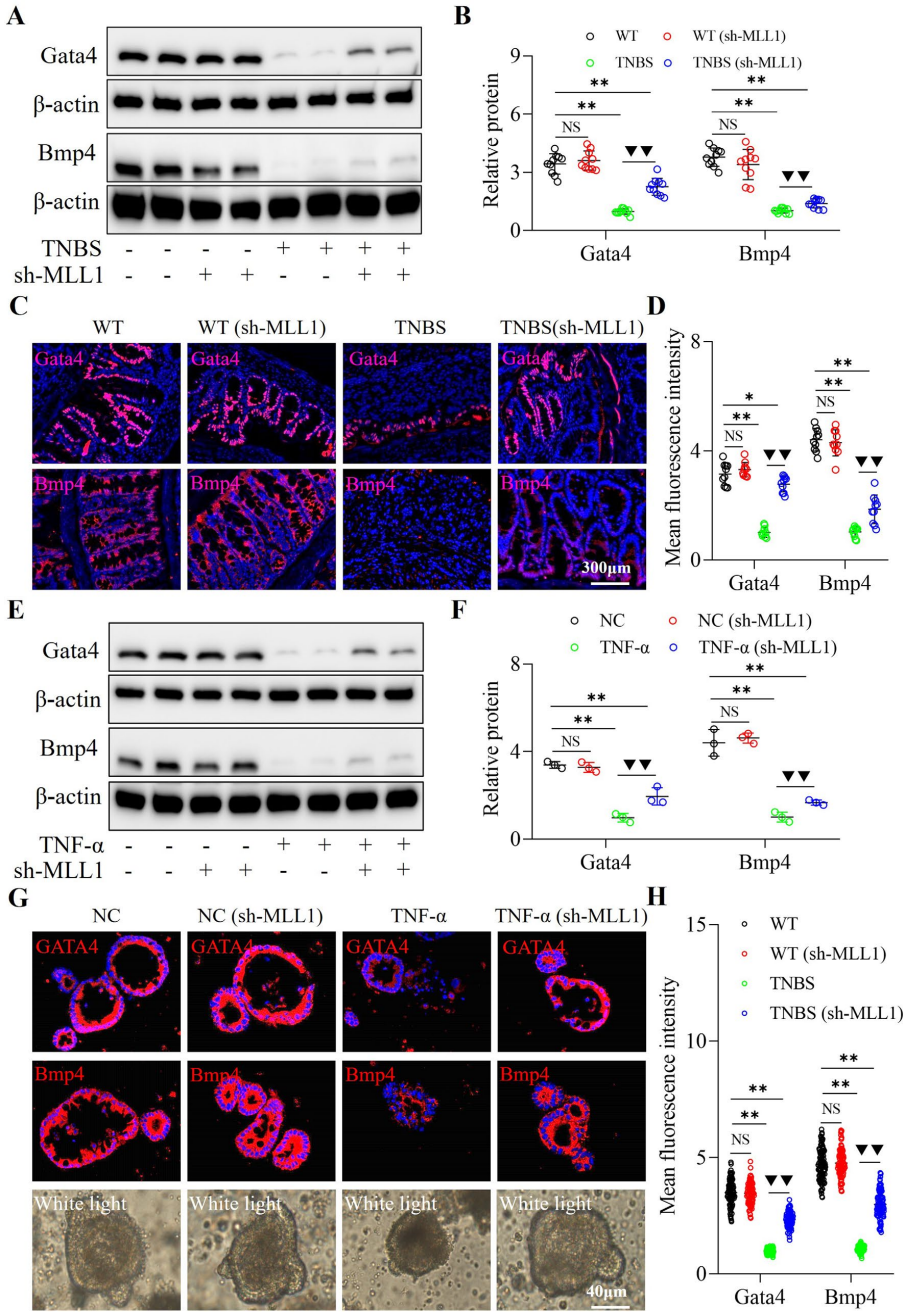
MLL1 inhibition activates Gata4/Bmp4 signalling axis. (A and B) Phosphorylated Gata4/Bmp4 levels by immunoblotting (*n* = 10/group). (C and D) Gata4/Bmp4 subcellular localisation via fluorescence microscopy (*n* = 10/group). (E and F) Gata4/Bmp4 protein quantitation in colonoids (*n* = 3/group). (G and H) Intracellular Gata4/Bmp4 levels in colonoids via fluorescence microscopy (*n* = 10/group). Data: Mean ± SD; *p < 0.05 versus NC/WT; ^▼^ *p* < 0.05 versus TNF‐α/TNBS; **p < 0.0001 versus NC/WT groups; ^▼▼^ *p* < 0.0001 versus TNF‐α/TNBS; NS: No significance.

Recent studies have shown that MLL1 suppresses IEC differentiation by inhibiting Gata4/Bmp4 signalling pathway activation [21]. To support MLL1 regulating the GATA4/BMP4 axis epigenetically, we established MLL1/GATA4 single/double‐knockdown NCM460 cells. MLL1 depletion reduced global H3K4me3 and its GATA4 promoter occupancy but elevated local H3K4me3, offering direct epigenetic evidence (Figure S6E–I). By inhibiting the expression of MLL1 in organoids, it was found that there is an interaction between MLL1 and GATA4 (Figure S6J). Western blot analysis and immunofluorescence confirmed that MLL1 regulates the Gata4/Bmp4 signalling axis. Specifically, MLL1 inhibition increased the protein levels of both Gata4 and Bmp4, as observed in two experimental settings: TNBS‐induced colitis models (Figure 7A–D) and TNF‐α‐stimulated intestinal organoids (Figure 7E–H). These findings suggest that MLL1 may suppress IEC differentiation by impairing the function of the Gata4/Bmp4 signalling axis.

### 3.8 Inhibition of the Pro‐Differentiation Effects of MLL1 Was Reversed by Inhibiting the Gata4/Bmp4 Signalling Pathway

**FIGURE 8:**
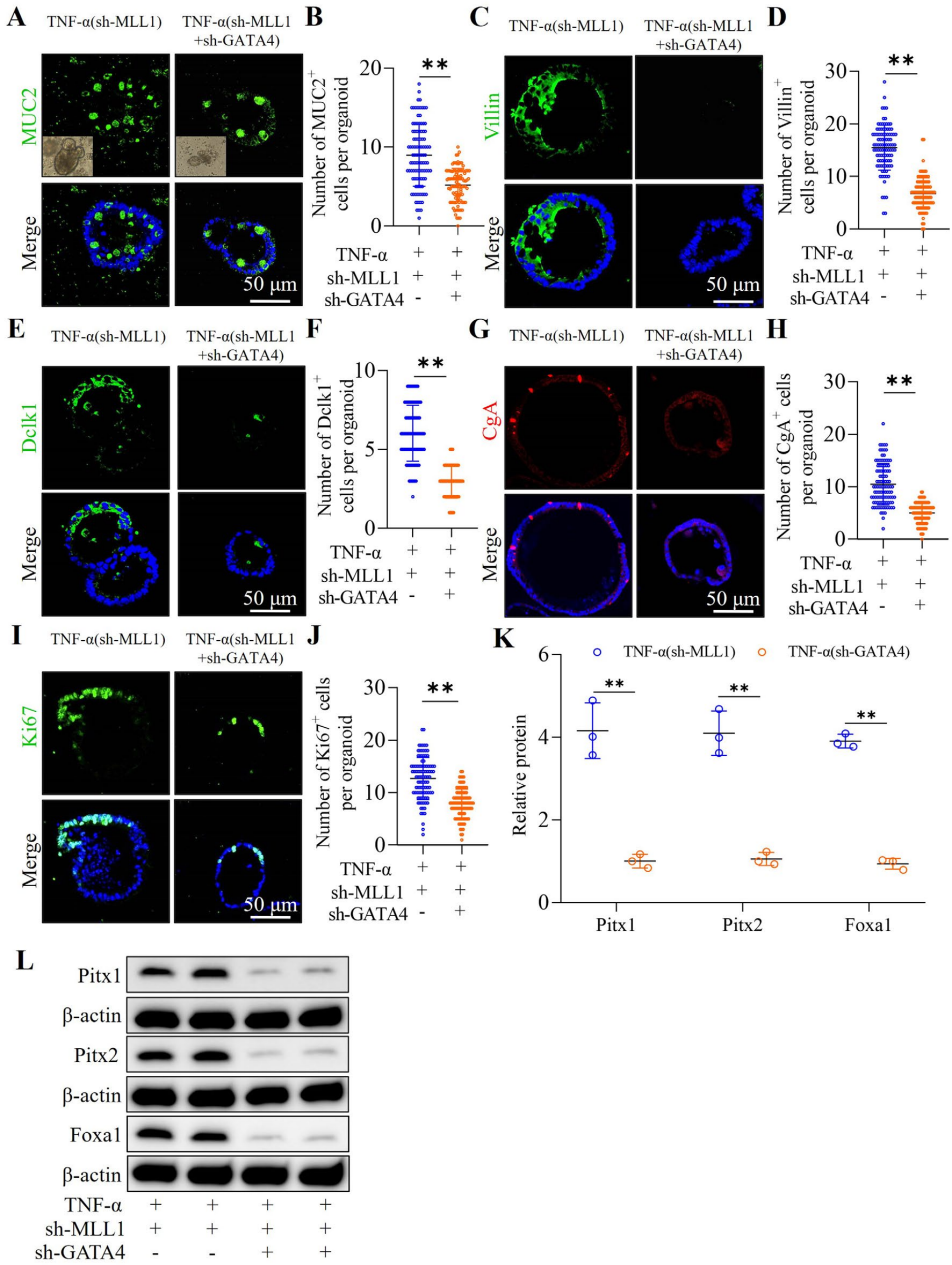
Gata4 signalling mediates MLL1‐driven differentiation. (A and B) The mean fluorescence intensity of goblet cell post‐Gata4 inhibition, in the bottom right corner is a representative white light microscopy image of organoids (*n* = 100/group). (C and D) The mean fluorescence intensity of absorptive cell densities under dual MLL1/Gata4 suppression (*n* = 100/group). (E and F) The mean fluorescence intensity of tuft cell populations following combinatorial knockdown (*n* = 100/group). (G and H) CgA^+^ enteroendocrine cell frequencies (*n* = 100/group). (I and J) Ki67^+^ TA cell enumeration (*n* = 100/group). (K and L) Pitx1/Pitx2/Foxa1 protein dynamics (*n* = 3/group). TNF‐α‐shMLL1 (AAV‐mediated MLL1 knockdown + TNF‐α), TNF‐α‐shGATA4 (lentiviral MLL1 + GATA4 suppression + TNF‐α). Data: Mean ± SD; *p < 0.05 versus TNF‐α‐shMLL1, **p < 0.0001 versus TNF‐α‐shMLL1.

To determine the direct role of MLL1 in promoting differentiation via the Gata4/Bmp4 pathway in IECs, we investigated the effects of GATA4 on organoids in the TNF‐α (sh‐MLL1) group. Immunofluorescence analysis delineated lineage‐specific effects of GATA4 modulation in TNF‐α‐treated organoids. shRNA‐mediated GATA4 knockdown (sh‐GATA4) substantially decreased goblet cell populations relative to sh‐MLL1 controls (Figure 8A,B). This suppression effect extended to EAC (Figure 8C,D), tuft cells (Figure 8E,F) and enteroendocrine cells (Figure 8G,H) in comparative analyses. Quantitative assessments further demonstrated diminished TA cell quantities in GATA4‐inhibited organoids versus MLL1‐silenced counterparts (Figure 8I,J). Complementary western blot analyses revealed restoration of Pitx1, Pitx2 and Foxa1 protein levels upon GATA4 blockade (Figure 8K,L), counteracting the expression patterns induced by MLL1 downregulation. These coordinated observations establish functional linkage between Gata4/Bmp4 pathway activity and MLL1‐driven differentiation constraints.

### 3.9 Inhibition of the Gata4/Bmp4 Signalling Pathway Reverses the Protective Effect of MLL1 Downregulation

**FIGURE 9:**
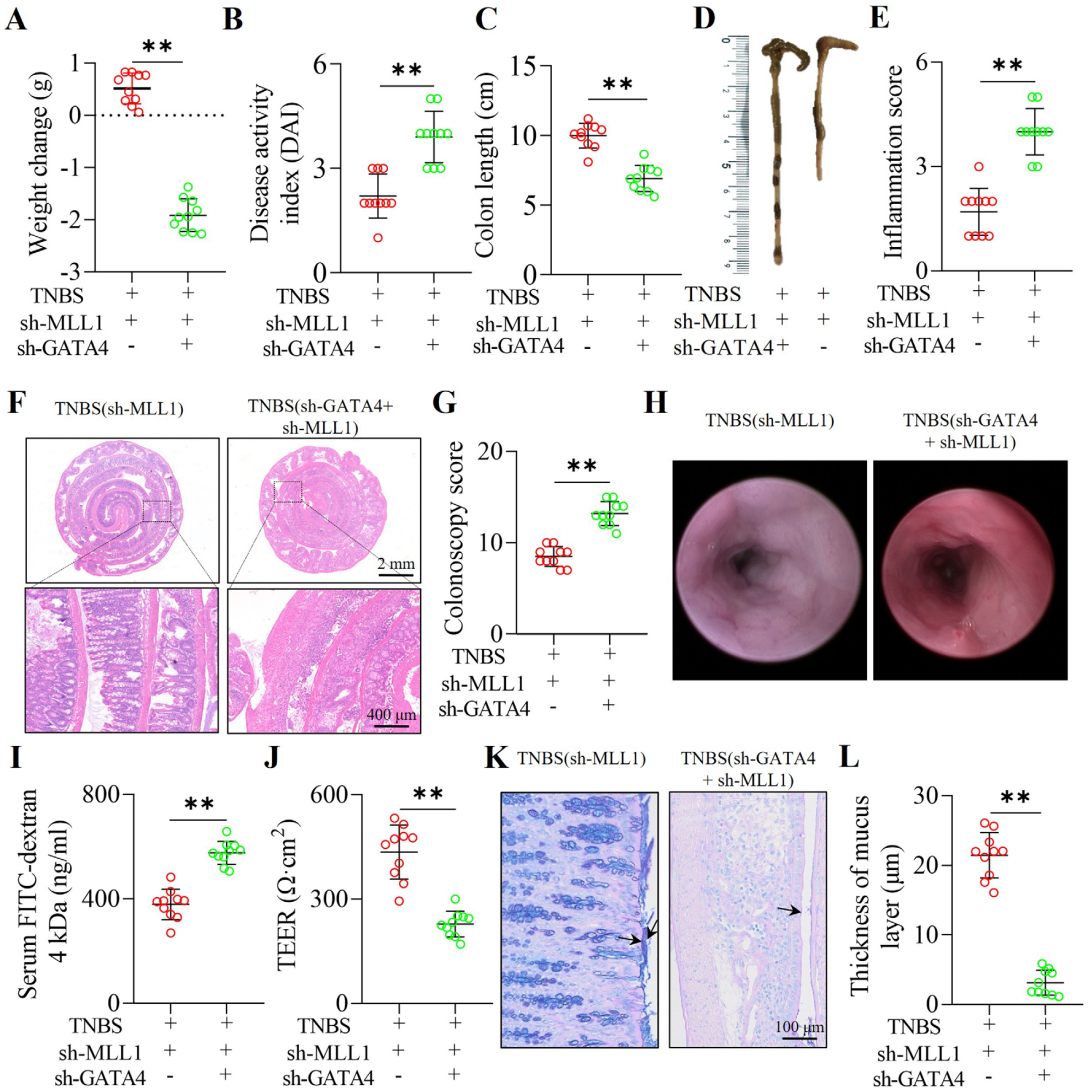
Gata4 blockade attenuates MLL1‐mediated colitis protection. (A) Body weight trajectories (*n* = 10/group). (B) DAI scoring (*n* = 10/group). (C and D) Colon length measurements (*n* = 10/group). (E) Histopathological inflammation grading (*n* = 10/group). (F) Representative HE‐stained sections. (G and H) Endoscopic severity scores (*n* = 10/group). (I) Serum FITC‐dextran levels (*n* = 10/group). (J) Colonic TEER values (*n* = 10/group). (K and L) PAS‐based mucus thickness analysis (*n* = 10/group). TNBS‐shMLL1 (AAV‐MLL1 knockdown + TNBS), TNBS‐shGATA4 (AAV‐MLL1 + shGATA4 + TNBS). Data: Mean ± SD; *p < 0.05 versus TNBS‐shMLL1, **p < 0.0001 versus TNBS‐shMLL1.

To investigate whether MLL1 exerts a protective effect through the Gata4/Bmp4 pathway, we analysed the impact of Gata4 on TNBS‐induced colitis mice that had been administered AAV‐shRNA‐MLL1. Compared with the TNBS (sh‐MLL1) group, the TNBS (sh‐MLL1 + sh‐Gata4) group showed significantly more severe weight loss (Figure 9A) and a higher DAI score (Figure 9B). These initial observations suggested that Gata4 inhibition might exacerbate colitis in the context of MLL1 silencing. Further analyses confirmed this trend: genetic ablation of GATA4 in MLL1‐silenced TNBS‐colitis mice (via AAV‐shRNA‐MLL1) significantly augmented inflammatory manifestations. This was evidenced by accelerated colonic shortening (Figure 9C,D), elevated histopathological indices (Figure 9E,F), and higher endoscopic ulceration scores (Figure 9G,H). Beyond inflammation, GATA4 inhibition in the sh‐Gata4 cohort also induced intestinal barrier dysfunction compared to TNBS (sh‐MLL1) controls, as indicated by elevated serum FITC‐dextran levels (Figure 9I) and reduced TEER (Figure 9J). Importantly, PAS‐stained histological analysis further confirmed defective mucus layer regeneration in GATA4‐deficient mice after AAV treatment (Figure 9K,L), which is critical for maintaining intestinal homeostasis. Collectively, these findings demonstrate that decreased MLL1 expression exerts a protective effect by inhibiting the activation of the Gata4/Bmp4 signalling pathway.

## 4 Discussion

Elevated MLL1 protein levels were detected in inflamed intestinal mucosa of CD patients and experimental TNBS‐colitis murine models. Genetic or pharmacological suppression of MLL1 ameliorated intestinal inflammation through activation of the Gata4/Bmp4 signalling axis, which enhanced mucosal regeneration by restoring TJ architecture and goblet cell differentiation.

MLL1 is a histone H3 lysine 4 (H3K4) methyltransferase that is essential for regulating the expression of specific genes [50]. Murine models subjected to ischaemia‐reperfusion injury exhibited marked MLL1 overexpression, concomitant with expanded H3K4me3^+^ cell populations within pathologically altered tissue niches [51]. Comparative analysis demonstrated upregulated MLL1 in colonic biopsies from CD patients and murine TNBS‐colitis models relative to healthy counterparts. Notably, renal pathology assessments revealed epithelial cell‐specific MLL1 enrichment following injury induction [52]. Subsequent analysis identified epithelial compartment‐specific MLL1 dysregulation, manifesting as pathological overexpression in both clinical Crohn's specimens and experimental colitis systems. Conversely, under normal physiological conditions, MLL1 expression was limited to ISCs and TA cells, and MLL1 expression was absent in intestinal villi. The abnormal expression and distribution of MLL1 may indicate pathology in CD colitis. However, the specific function of MLL1 in CD‐like colitis remains unclear.

A previous study revealed that MLL1 gene knockdown in mice did not induce retinopathy, whereas null mutations in MLL1 inhibited tumour formation without affecting normal tissue morphology [53]. Additionally, no significant increase in the DAI index was noted following MLL1 knockdown, suggesting that this therapeutic approach is potentially safe. Pathological analyses and colonoscopy scores revealed that MLL1 downregulation effectively mitigated intestinal inflammation in mice, similar to its role in ameliorating renal fibrosis and inflammation induced by ischaemia–reperfusion [51, 54]. Recent research has demonstrated that MLL1 plays a role in modulating the expression of genes downstream of NF‐κB [55], and ELISA and PCR data further suggest that the inhibition of MLL1 can decrease the expression of inflammatory mediators. These data suggest that reducing MLL1 expression could alleviate CD‐like colitis symptoms, but further research is needed to understand the mechanisms underlying its protective effects.

The restoration of intestinal barrier structure and function has been shown to be advantageous for individuals with CD enteritis [56]. Functional validation revealed MLL1 suppression preserves mucosal barrier integrity in experimental colitis contexts, including both TNBS‐challenged murine models and TNF‐α‐treated intestinal organoids. Emerging studies highlight that dysregulation of TJ components, including ZO1 and Claudin1, disrupts TJ architecture and compromises intestinal barrier homeostasis [57]. Molecular imaging and protein profiling revealed that MLL1 knockdown dynamically regulates ZO1/Claudin1 expression, potentially offsetting MLL1‐mediated spatial deficits in TJ organisation. Reducing MLL1 expression seems to increase intestinal barrier integrity, thereby ameliorating the symptoms of CD‐like colitis.

Emerging evidence indicates that promoting IEC maturation can effectively repair the intestinal barrier [58]. MLL1, which is part of the conserved SET1 gene family, is essential for regulating various developmental regulatory genes in animals [59]. The knockdown of MLL1 has been shown to upregulate neural markers and induce the differentiation of neuron‐like cells [60]. Moreover, inhibitors of the MLL1 pathway, such as MM408 and MI503, increase the efficiency of fibroblast conversion into functional cardiomyocyte‐like cells [61]. Our findings indicate that downregulation of MLL1 expression leads to increases in absorptive, goblet and tuft cell numbers in the colons and organoids from TNBS model mice. MLL1 is known to be expressed in ISCs and TA cells, and its deletion is correlated with increases in the numbers of goblet cells and a propensity for secretory lineage differentiation [47]. Immunofluorescence analysis indicated that MLL1 inhibition led to an increase in Ki67^+^ cell numbers without affecting Olfm4^+^ cell numbers; these results suggest that cellular differentiation capacity was improved. This indicates that MLL1 may exert its function by regulating other specific genes or signalling pathways: MLL1 might not directly regulate Olfm4 expression but instead indirectly affect the function of ISCs through the regulation of other genes or signalling pathways [47]. Olfm4 is a gene highly expressed in ISCs and is often used as a marker for ISCs [62]. Although Olfm4‐positive cells are an important component of ISCs, the maintenance and function of stem cells are regulated by multiple factors [63]. MLL1 may influence the function of ISCs by regulating other key genes or signalling pathways, such as the Wnt signalling pathway, rather than directly affecting the number of Olfm4‐positive stem cells [64]. MLL1 is essential for the upregulation of various intestinal transcription factors, such as Pitx1, Pitx2 and Foxa1 [65]. Immunoblot quantification established that genetic perturbations of MLL1 dynamically control the proteostatic landscape of key transcription regulators (Pitx1/Pitx2/Foxa1). Mechanistically, MLL1 acts as a master epigenetic coordinator driving mucosal restitution through precision modulation of enterocytic lineage specification, though comprehensive delineation of downstream molecular transducers orchestrating chromatin‐cytoskeletal crosstalk remains an imperative research frontier.

Histological analysis of Wnt‐mutant intestinal tumour‐initiating cells revealed that MLL1 regulates the transcription factor Gata4, which is involved in cuprocyte differentiation [66]. Our research results indicate that reduced expression of MLL1 leads to increased Gata4 expression in the colon and organoids of TNBS model mice. Previous studies have demonstrated that inhibition of GATA4 expression in mice results in decreased protein levels of BMP4, consequently impairing odontogenic/osteogenic differentiation [67]. Subsequent examination demonstrated that downregulation of MLL1 expression resulted in decreased levels of GATA4 and BMP4. BMP4 facilitates cardiomyogenic development, promotes differentiation by inhibiting mesenchymal stem cell characteristics and notably, elevates the expression of Gata4, thereby inducing the upregulation of Bmp4 and the secretory differentiation marker Muc2 [68]. Integrative translational models (organoid‐based assays coupled with transgenic murine systems) demonstrated that combinatorial MLL1 knockdown and Gata4 pharmacological ablation dose‐dependently reversed therapeutic barrier restoration. We also found that after knocking down GATA4 expression in organoids, the expression levels of MUC2 and ZO1 decreased (Figure S6C,D), suggesting that MUC2 and ZO1 might be downstream effectors of Gata4/Bmp4. This mechanistically positions MLL1 as a central epigenetic conductor orchestrating mucosal lineage commitment via a novel transcriptional‐structural axis converging on GATA4‐mediated BMP morphogen regulation.

Our study revealed that inhibiting MLL1 activity activates the Gata4/Bmp4 signalling pathway, promoting IEC differentiation and increasing intestinal barrier repair efficiency. These findings delineate novel pathophysiological dimensions of MLL1 in gastrointestinal homeostasis. Mechanistic dissection revealed that MLL1‐dependent chromatin remodelling mitigates TNBS‐induced colitis progression through potentiation of epithelial barrier integrity. Aligning with the established centrality of mucosal repair mechanisms in inflammatory bowel disease, our data nominate MLL1 as a molecularly tractable target for CD therapeutic development.

There are acknowledged limitations to our study. This study directly demonstrates the autonomous regulation of MLL1 on IECs through in vitro pure epithelial organoid experiments, combined with specific changes in epithelium‐specific phenotypes in in vivo experiments, which have sufficiently excluded immune cell‐mediated indirect effects. The epithelium‐specific knockout model can serve as a supplement for subsequent research (such as exploring segment‐specific effects in different parts of the intestine), but it is not necessary for validating the core mechanism at present. The role of MLL1 in ISC differentiation is complex and multifaceted, influenced by various factors. While MLL1 plays a role in maintaining ISC stemness, under specific conditions, knocking down MLL1 can actually promote the differentiation of epithelial cells [69]. This seemingly contradictory phenomenon suggests that further research is needed to deeply understand the interactions between MLL1 and other signalling pathways (e.g., Wnt and Notch signalling pathway), epigenetic modifiers and the intestinal microenvironment, to fully comprehend the role of MLL1 in intestinal homeostasis and disease. Although the TNBS‐induced mouse model is a classic and widely used system for investigating CD pathogenesis, it is notably limited in its capacity to replicate the chronic inflammatory cascades and tissue pathological changes observed in clinical colitis. Additionally, there are many mechanisms by which MLL1 hinders the amelioration of colitis, and these mechanisms may include interference with macrophage inflammation and antiapoptotic pathways; thus, further investigation in future studies is necessary. Furthermore, MLL1 may modulate multiple signalling pathways, potentially impacting IEC differentiation through alternative pathways. To gain a deeper understanding of the interaction between MLL1 and the Gata4/Bmp4 signalling pathway at the chromatin level, future research could utilise techniques such as ChIP‐seq, RNA sequencing and CRISPR/Cas9 to analyse MLL1 binding sites, changes in gene expression profiles, and the synergistic mechanisms with other factors, thereby revealing regulatory molecular mechanisms and providing new approaches for disease treatment.

An initial study indicated that reducing MLL1 expression alleviated CD‐like colitis in mice. MLL1 may protect intestinal barrier integrity by promoting IEC differentiation, potentially through Gata4/Bmp4 pathway activation. Given the regulatory effect of MLL1 on the differentiation capacity of IECs, MLL1 could hold promise in the treatment of individuals with CD.

Supplemental text and figures for this article include experimental details, raw data sets and figures that further support the key findings presented in the main text.

## Author Contributions

X.S., J.L., Y.C. and J.H. contributed to the study design, experiments and manuscript drafting. B.L., Y.Y., X.G., X.Z., L.T., Z.G. and L.Z. contributed to the experiments and data analysis. Y.W. and L.W. supervised the study, contributed to the critical revision of the manuscript and provided important intellectual content. All authors read and approved the final manuscript.

## Funding

This work was supported partially by funding from the National Natural Science Foundation of China (82070561, 82370534), the Anhui Province University Outstanding Youth Research Project (2022AH030138, 2022AH020085, 2023AH010067, 2023AH040289), the Anhui Province Clinical Medical Research Translation Programme (202427b10020088, 202427b10020094), the Outstanding Young Teachers Cultivation Programme of Anhui Provincial Education (YQYB2024042, YQYB2023019) and the Anhui Provincial Health and Health Commission Research Project (AHWJ2024Aa10051).

## Conflicts of Interest

The authors declare no conflicts of interest.

## Supporting information


**Figure S1:** Analysis of single‐cell sequencing results from the GEO database shows the expression of MLL1 in colon epithelial cells of CD patients. (A) t‐SNE dimensionality reduction plot, showing the distribution of different cell types in two‐dimensional space, with different colours representing different cell types; (B) stacked bar chart, presenting the proportion of each cell type in different samples, with different colours corresponding to different cell types; (C) expression levels of the KMT2A/MLL1 gene across different cell types and sample groups (CD and nIBD), CD (Crohn's disease), nIBD (no IBD); (D) analysis of the correlation between KMT2A/MLL1 and the gene set of inflammatory response signalling pathways, with the x‐axis representing the HALLMARK inflammatory response signalling pathway gene set (https://www.gsea‐msigdb.org/gsea/msigdb/cards/HALLMARK _INFLAMMATORY_RESPONSE). (E) MLL1 localisation via fluorescence microscopy.
**Figure S2:** AAV‐mediated MLL1 knockdown validation. (A and B) MLL1 protein levels of mice colon mucosa by immunoblotting. (C) MLL1 mRNA of mice colon mucosa quantitation via qRT‐PCR. (D) Disease activity index (DAI) assessment. Groups: WT, WT‐shMLL1, WT‐E‐AAV (empty vector), TNBS, TNBS‐E‐AAV (*n* = 10). Data: mean ± SD; *p < 0.0001 versus WT; NS: no significance, WT (sh‐MLL1) versus WT, ns: no significance, TNBS (E‐AAV) versus TNBS.
**Figure S3:** Therapeutic efficacy of MLL1 suppression in IL‐10^−/−^ mice. (A) Body weight trajectories across experimental groups. (B) Disease activity index (DAI) scoring. (C and D) Colon length measurements. (E and F) Endoscopic severity grading of colonic inflammation. (G) Representative H&E‐stained colonic sections. (H) Histopathological inflammation scores. (I and J) ZO1/Claudin1 localisation via fluorescence microscopy. WT (Untreated WT), WT‐shMLL1 (AAV‐mediated MLL1 knockdown), IL‐10^−/−^ colitis, IL‐10^−/−^‐shMLL1 (*n* = 10/group). Data: mean ± SD; *p < 0.05 versus WT; ^▼^ *p* < 0.05 versus IL‐10^−/−^; **p < 0.0001 versus WT groups; ^▼▼^ *p* < 0.0001 versus IL‐10^−/−^; NS: no significance, WT (sh‐MLL1) versus WT.
**Figure S4:** Intestinal stem cell dynamics post‐MLL1 suppression. (A and B) Analysis of the average numbers of stem cell markers (Olfm4) in the colon tissue of mice using fluorescence microscopy. WT, WT‐shMLL1, TNBS, TNBS‐shMLL1 (*n* = 10). Data: mean ± SD; **p < 0.0001 versus WT; NS: no significance, WT (sh‐MLL1) versus WT.
**Figure S5:** AAV‐mediated MLL1 knockdown in colonoids. (A and B) Quantitative analysis of MLL1 protein in mouse colon organoids. (C) Quantitative analysis of MLL1 mRNA in mouse colon organoids. (D) Intraluminal FITC‐dextran accumulation. (E) TEER measurements in colonoid cultures. (F) Monitor TEER values daily to determine the establishment of the colon organoid intestinal barrier model. NC, NC (sh‐MLL1), NC (E‐LV), TNF‐α, TNF‐α (E‐LV). Data: mean ± SD; *p < 0.0001 versus NC; NS: no significance.
**Figure S6:** Stem cell homeostasis in MLL1‐modulated colon organoids. (A and B) Analysis of the average fluorescence density of stem cell markers (Olfm4) in the mouse colon organoids using fluorescence microscopy. (C and D) GATA4, MUC2 and ZO1 protein quantitation in the mouse colon organoids. (E and F) H3K4me3, MLL1 and GATA4 protein quantitation in the NCM460 cells. (G and H) ChIP‐qPCR analysis: the region where MLL1 occupies on the GATA4 gene. Control IgG sample: 0.0001%–0.004% input. (I) MLL1 and GATA4 mRNA quantitation in the NCM460 cells. (J) ChIP analysis: investigating the interaction between MLL1 and GATA4 by inhibiting the expression of MLL1 in organoids. Data: mean ± SD; **p < 0.0001 versus NC; NS: no significance, versus NC.

**Table S1:** The general clinical information of patients involved in this study.

## Data Availability

The data that support the findings of this study are available on request from the corresponding author. The data are not publicly available due to privacy or ethical restrictions.
