# Supplementary material for: Downregulation of MLL1 Promotes Intestinal Epithelial Barrier Repair Through Gata4/Bmp4 Activation to Ameliorate Crohn's Disease‐Like Colitis in Mice
Source: Cell Prolif. 2026 Feb 24;59(7):e70182. doi: 10.1111/cpr.70182 (PMC13325501; doi:10.1111/cpr.70182)
Supplement: Supplementary file 1 — Figure S1: Analysis of single‐cell sequencing results from the GEO database shows the expression of MLL1 in colon epithelial cells of CD patients. (A) t‐SNE dimensionality reduction plot, showing the distribution of different cell types in two‐dimensional space, with different colours representing different cell types; (B) stacked bar chart, presenting the proportion of each cell type in different samples, with different colours corresponding to different cell types; (C) expression levels of the KMT2A/MLL1 gene across different cell types and sample groups (CD and nIBD), CD (Crohn's disease), nIBD (no IBD); (D) analysis of the correlation between KMT2A/MLL1 and the gene set of inflammatory response signalling pathways, with the x‐axis representing the HALLMARK inflammatory response signalling pathway gene set (https://www.gsea‐msigdb.org/gsea/msigdb/cards/HALLMARK _INFLAMMATORY_RESPONSE). (E) MLL1 localisation via fluorescence microscopy. Figure S2: AAV‐mediated MLL1 knockdown validation. (A and B) MLL1 protein levels of mice colon mucosa by immunoblotting. (C) MLL1 mRNA of mice colon mucosa quantitation via qRT‐PCR. (D) Disease activity index (DAI) assessment. Groups: WT, WT‐shMLL1, WT‐E‐AAV (empty vector), TNBS, TNBS‐E‐AAV (n = 10). Data: mean ± SD; *p < 0.0001 versus WT; NS: no significance, WT (sh‐MLL1) versus WT, ns: no significance, TNBS (E‐AAV) versus TNBS. Figure S3: Therapeutic efficacy of MLL1 suppression in IL‐10−/− mice. (A) Body weight trajectories across experimental groups. (B) Disease activity index (DAI) scoring. (C and D) Colon length measurements. (E and F) Endoscopic severity grading of colonic inflammation. (G) Representative H&E‐stained colonic sections. (H) Histopathological inflammation scores. (I and J) ZO1/Claudin1 localisation via fluorescence microscopy. WT (Untreated WT), WT‐shMLL1 (AAV‐mediated MLL1 knockdown), IL‐10−/− colitis, IL‐10−/−‐shMLL1 (n = 10/group). Data: mean ± SD; *p < 0.05 versus WT; ▼ p < 0.05 versus IL‐10−/−; **p < 0.000 [file CPR-59-e70182-s001.docx]

# **Supplementary figures and figure legends**


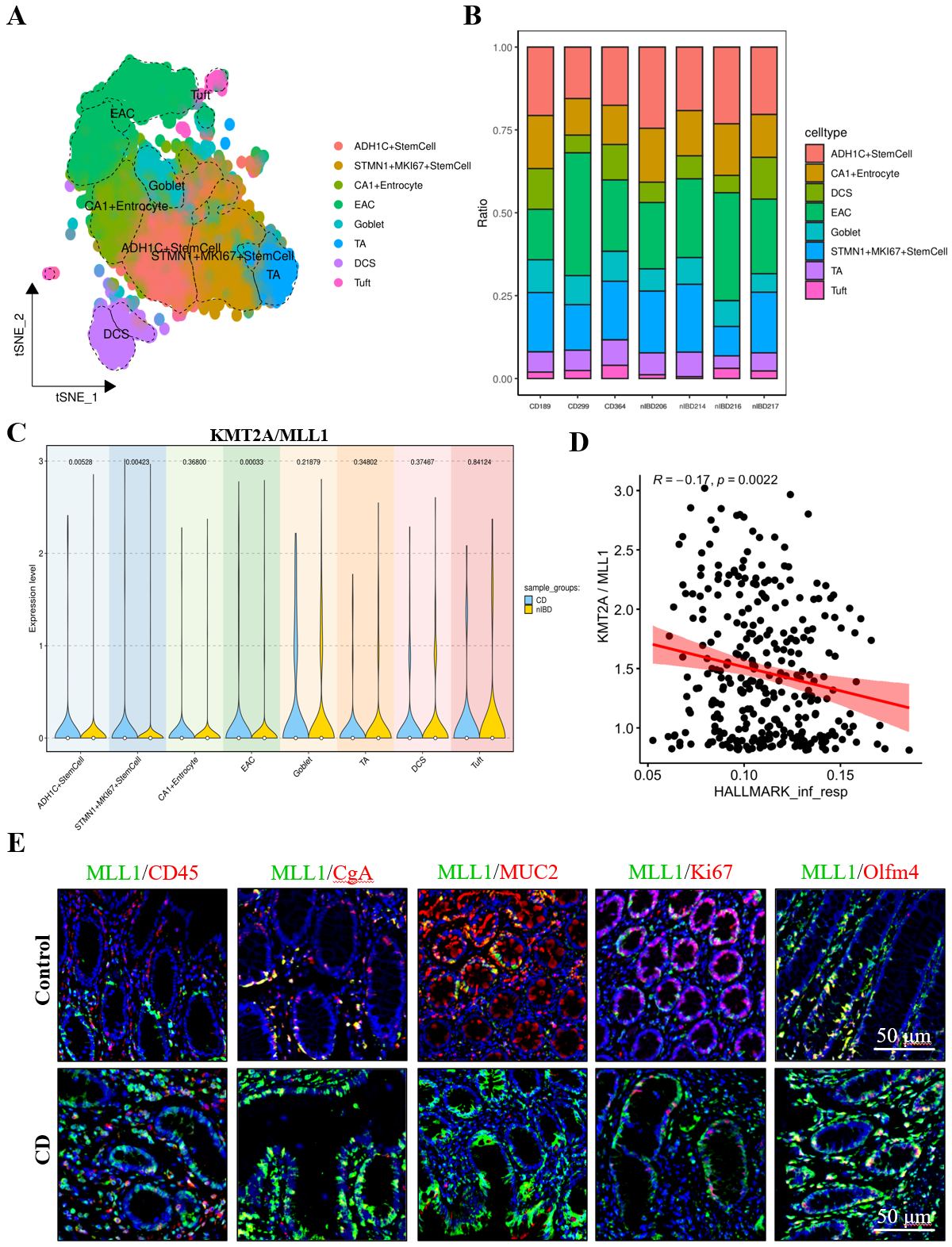


***Figure S1 Analysis of single-cell sequencing results from the GEO database shows the expression of MLL1 in colon epithelial cells of CD patients.***

(A) t-SNE dimensionality reduction plot, showing the distribution of different cell types in two-dimensional space, with different colors representing different cell types; (B) stacked bar chart, presenting the proportion of each cell type in different samples, with different colors corresponding to different cell types; (C) Expression levels of the KMT2A/MLL1 gene across different cell types and sample groups (CD and nIBD), CD (Crohn's disease), nIBD (no IBD); (D) Analysis of the correlation between KMT2A/MLL1 and the gene set of inflammatory response signaling pathways, with the x-axis representing the HALLMARK inflammatory response signaling pathway gene set (https://www.gsea-msigdb.org/gsea/msigdb/cards/HALLMARK _INFLAMMATORY_RESPONSE). (E) MLL1 localization via fluorescence microscopy.

1.
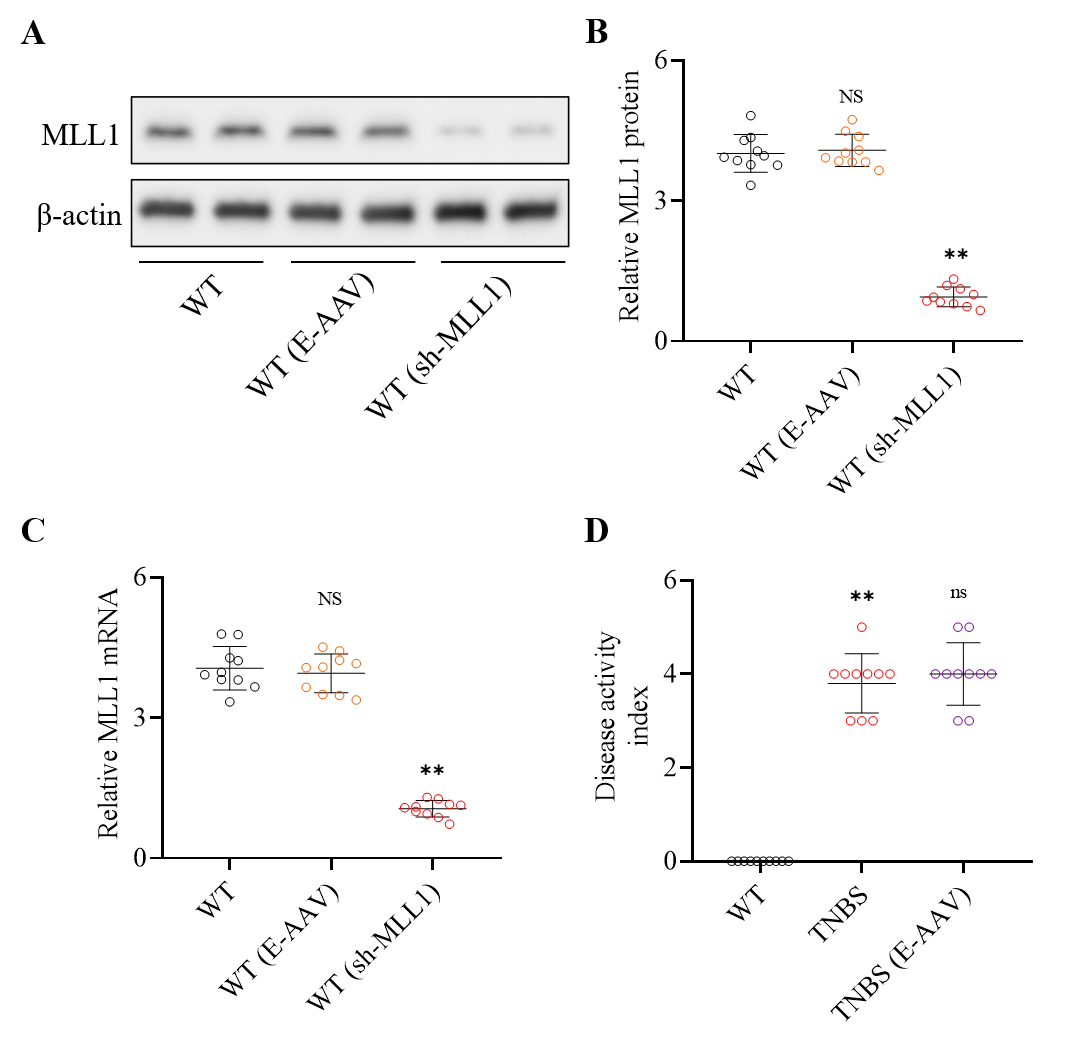


***Figure S2 AAV-Mediated MLL1 Knockdown Validation.***

(A-B) MLL1 protein levels of mice colon mucosa by immunoblotting. (C) MLL1 mRNA of mice colon mucosa quantitation via qRT-PCR. (D) Disease activity index (DAI) assessment. Groups: WT, WT-shMLL1, WT-E-AAV (empty vector), TNBS, TNBS-E-AAV (n=10). Data: mean ± SD; **P*<0.0001 vs. WT; NS: no significance, WT (sh-MLL1) vs. WT, ns: no significance, TNBS (E-AAV) vs. TNBS.


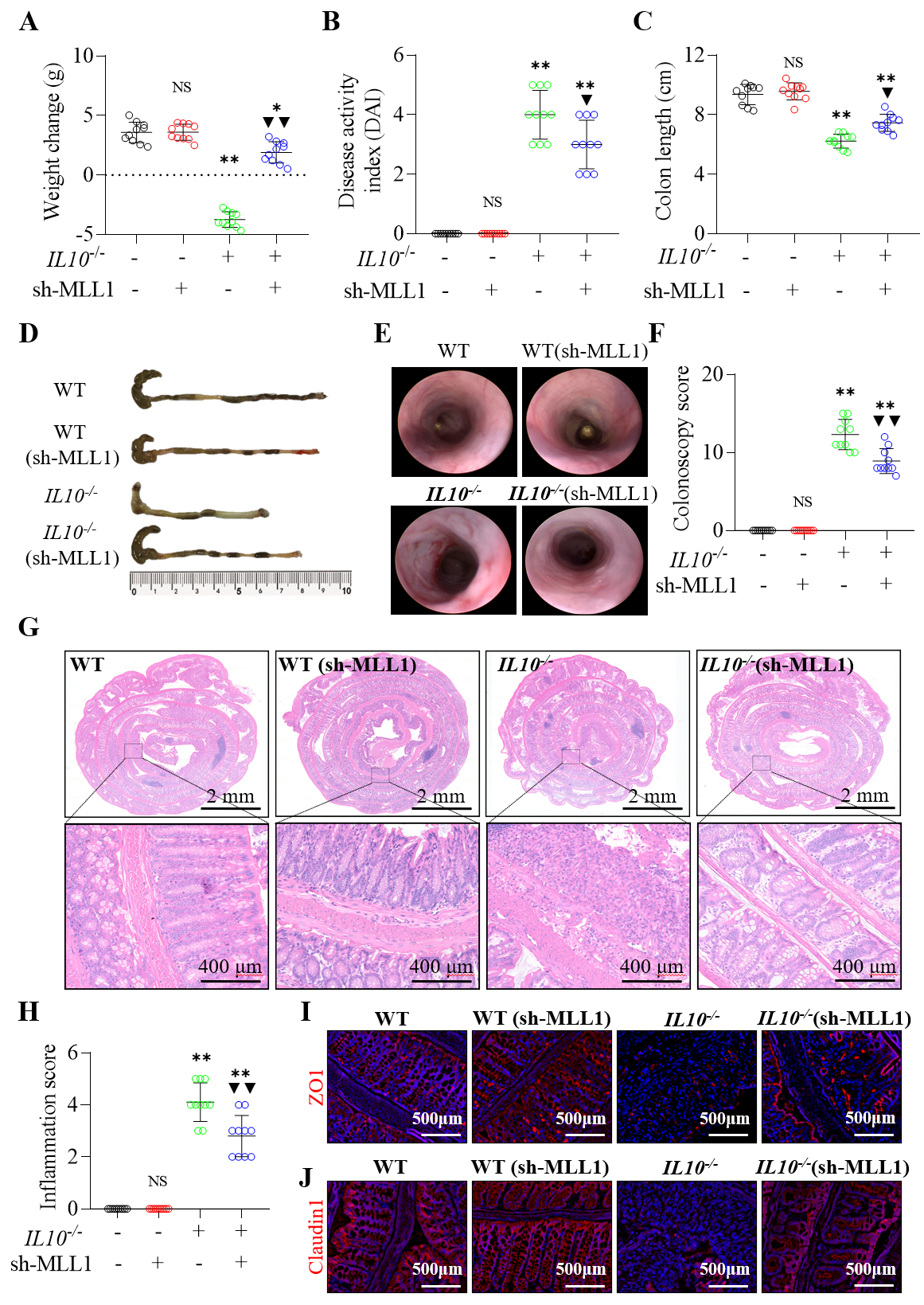


***Figure S3 Therapeutic Efficacy of MLL1 Suppression in*** ***IL-10^−/−^ mice.***

(A) Body weight trajectories across experimental groups. (B) Disease activity index (DAI) scoring. (C-D) Colon length measurements. (E-F) Endoscopic severity grading of colonic inflammation. (G) Representative H&E-stained colonic sections. (H) Histopathological inflammation scores. (I-J) ZO1/Claudin1 localization via fluorescence microscopy. WT (Untreated WT), WT-shMLL1 (AAV-mediated MLL1 knockdown), *IL-10^−/−^* colitis, *IL-10^−/−^*-shMLL1 (n=10/group). Data: mean ± SD; **P*<0.05 vs. WT; ^▼^*P*<0.05 vs. *IL-10^−/−^*; ***P*<0.0001 vs. WT groups; ^▼▼^*P*<0.0001 vs. *IL-10^−/−^*; NS: no significance, WT (sh-MLL1) vs. WT.


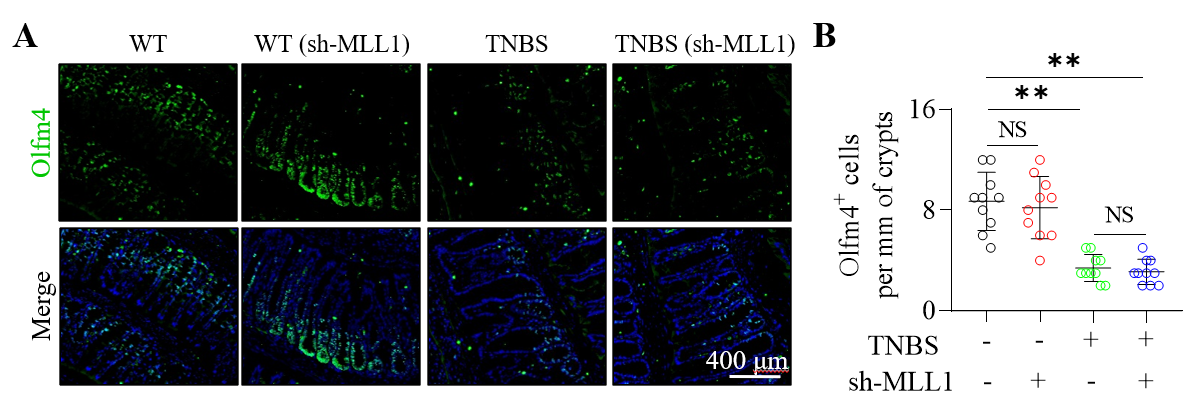


***Figure S4 Intestinal Stem Cell Dynamics Post-MLL1 Suppression.***

(A-B) Analysis of the average numbers of stem cell markers (Olfm4) in the colon tissue of mice using fluorescence microscopy. WT, WT-shMLL1, TNBS, TNBS-shMLL1 (n=10). Data: mean ± SD; ***P*<0.0001 vs. WT; NS: no significance, WT (sh-MLL1) vs. WT.


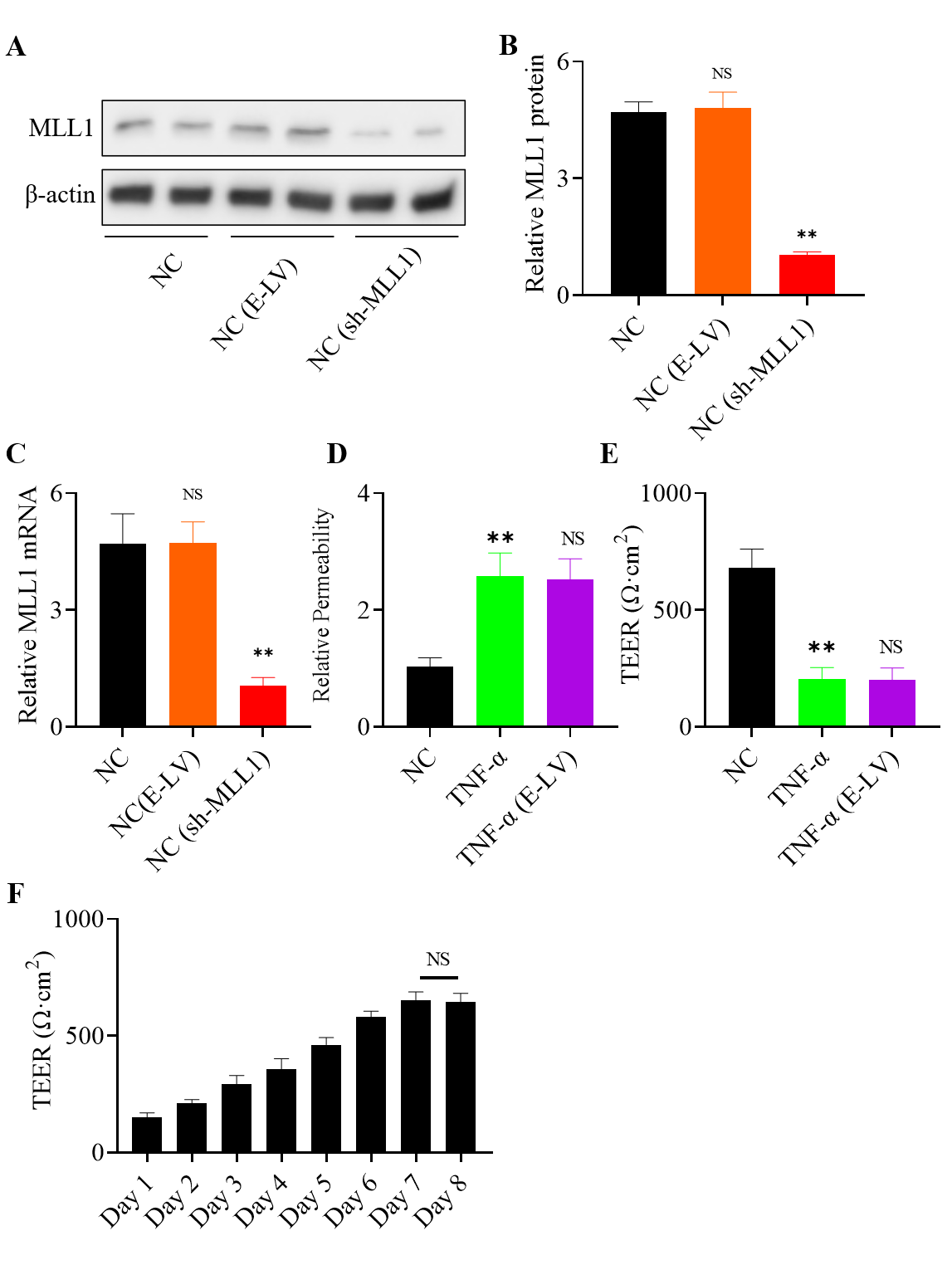


***Figure S5 AAV-Mediated MLL1 Knockdown in Colonoids.***

(A-B) Quantitative analysis of MLL1 protein in mouse colon organoids. (C) Quantitative analysis of MLL1 mRNA in mouse colon organoids. (D) Intraluminal FITC-dextran accumulation. (E) TEER measurements in colonoid cultures. (F) Monitor TEER values daily to determine the establishment of the colon organoid intestinal barrier model. NC, NC (sh-MLL1), NC (E-LV), TNF-α, TNF-α (E-LV). Data: mean ± SD; **P*<0.0001 vs. NC; NS: no significance.


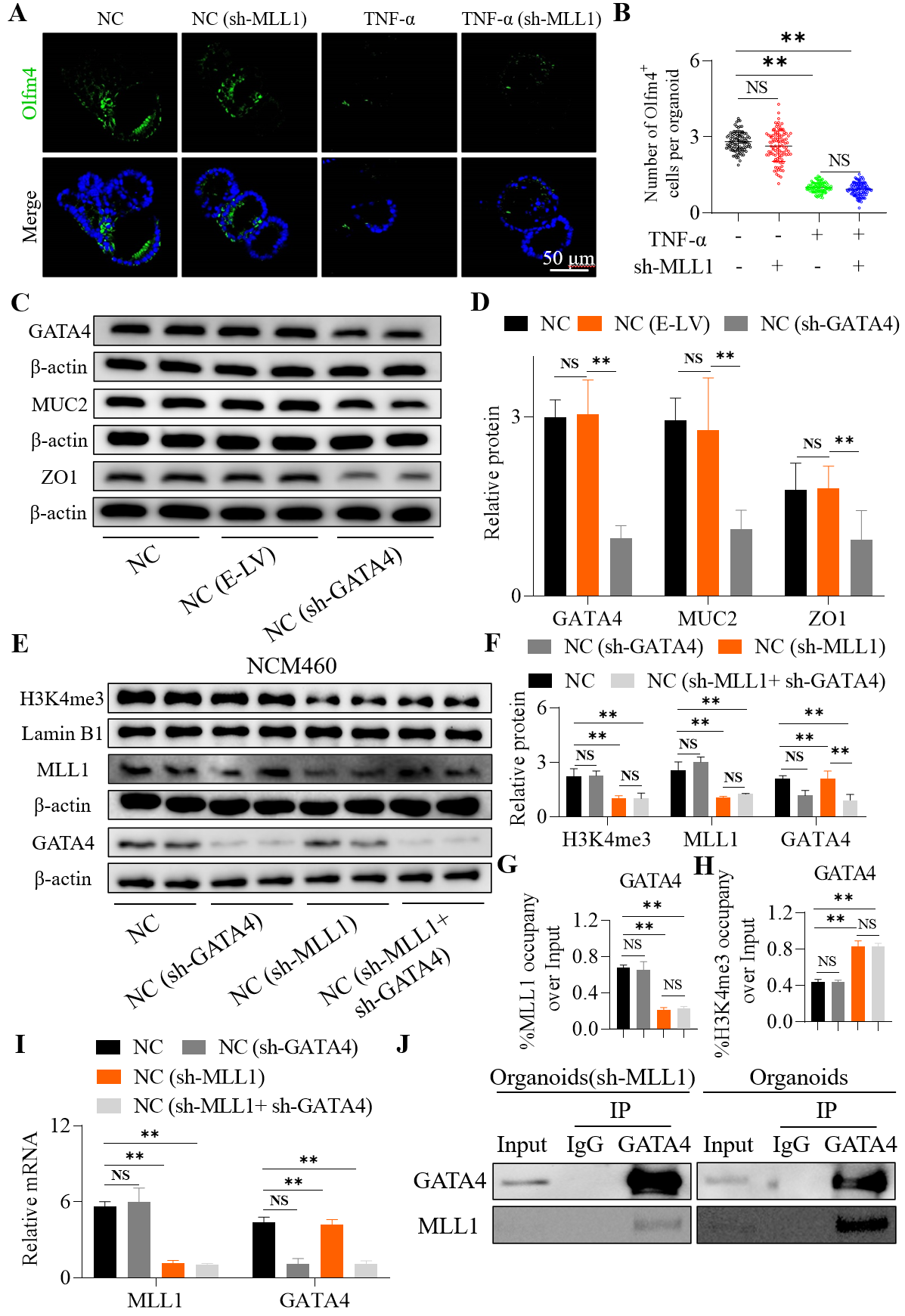


***Figure S6 Stem Cell Homeostasis in MLL1-Modulated Colon Organoids.***

(A-B) Analysis of the average fluorescence density of stem cell markers (Olfm4) in the mouse colon organoids using fluorescence microscopy. (C-D) GATA4, MUC2 and ZO1 protein quantitation in the mouse colon organoids. (E-F) H3K4me3, MLL1 and GATA4 protein quantitation in the NCM460 cells. (G-H) ChIP-qPCR analysis: the region where MLL1 occupies on the GATA4 gene. Control IgG sample: 0.0001-0.004% input. (I) MLL1 and GATA4 mRNA quantitation in the NCM460 cells. (J) ChIP analysis: investigating the interaction between MLL1 and GATA4 by inhibiting the expression of MLL1 in organoids. Data: mean ± SD; ***P*<0.0001 vs. NC; NS: no significance, vs. NC.
