# Supplementary material for: Downregulation of MLL1 Promotes Intestinal Epithelial Barrier Repair Through Gata4/Bmp4 Activation to Ameliorate Crohn's Disease‐Like Colitis in Mice
Source: Cell Prolif. 2026 Feb 24;59(7):e70182. doi: 10.1111/cpr.70182 (PMC13325501; doi:10.1111/cpr.70182)
Supplement: Supplementary file 2 — Table S1: The general clinical information of patients involved in this study. [file CPR-59-e70182-s002.docx]

# Supplementary Tables

## Supplementary Table 1. The general clinical information of patients involved in this study.

|  | **CD group**  **(n=13)** | **NL group**  **(n=14)** | ***P* value** |
| --- | --- | --- | --- |
| Sex (male, %) | 9 (69.23) | 7 (50.00) | 0.801 |
| BMI (kg/m^2^)* | 18.8±3.5 | 19.3 ± 2.6 | 0.672 |
| Smoking history (n/%) |  |  | 0.653 |
| Never | 11 (84.61) | 9 (64.29) | - |
| Former | 2 (15.38) | 5 (35.71) | - |
| Current | 0 (0.00) | 0 (0.00) | - |
| Age at surgery (y)* | 40.8±8.9 | 48.6 ± 7.0 |  |
| Disease location (n/%) |  | - | - |
| L1 (ileal) | 0 (0.00) | - | - |
| L2 (colonic) | 2 (15.38) | - | - |
| L3 (ileocolonic) | 10(84.61) | - | - |
| Disease behavior (n/%) |  | - | - |
| B1 (inﬂammatory) | 0 (0.00) | - | - |
| B2 (stricturing) | 11 (84.61) | - | - |
| B3 (penetrating) | 2 (15.38) | - | - |
| Perianal complications (n/%) | 2 (15.38) | - | - |
| Preoperative medication (n/%) | - | - | - |
| Azathioprine | 1 (7.69) | - | - |
| Infliximab | 0 (0.00) | - | - |
| Corticosteroids | 1 (7.69) |  |  |

*Data are expressed as the mean ± standard deviation (SD).
